# In Silico Analysis of Mechanisms of Maribavir-Induced Inhibition and Drug Resistance Mutations in pUL97 Kinase Structural Prediction with AlphaFold2

**DOI:** 10.3390/v17070941

**Published:** 2025-07-02

**Authors:** Jocelyne Piret, Guy Boivin

**Affiliations:** 1 Research Center of the CHU de Quebec-Laval University, Quebec City, QC G1V 4G2, Canada; 2 Department of Pediatrics, Faculty of Medicine, Laval University, Quebec City, QC G1V 0A6, Canada

**Keywords:** human cytomegalovirus, maribavir, ganciclovir, cyclopropavir, drug resistance, gatekeeper residue, pUL97 kinase, predicted protein structure, AlphaFold2, docking

## Abstract

Infections with cytomegalovirus (CMV) can result in increased morbidity and mortality in immunocompromised patients. The pUL97 kinase is a critical enzyme in the regulation of CMV replication. Although it does not phosphorylate deoxynucleosides, this enzyme is involved in the first phosphorylation step of ganciclovir (GCV), a viral DNA polymerase inhibitor. In contrast, maribavir (MBV) is a specific inhibitor of pUL97 kinase activity. In this paper, we analyzed the already-reported amino acid changes, conferring resistance to MBV and cross-resistance to GCV, in the pUL97 protein structure, predicted with AlphaFold2. Docking experiments suggest that MBV is a dual-site inhibitor, targeting ATP binding and substrate phosphorylation. Substitutions that confer resistance to MBV only may directly or indirectly alter the shape of the cavity in the vicinity of the invariant K355 in the putative ATP binding site, without affecting the viral growth. The most frequently encountered T409M substitution may correspond to a gatekeeper mutation. Substitutions that induce cross-resistance to MBV and GCV may directly or indirectly affect the environment of D456 and N461 residues in the catalytic loop, with reduced viral replicative capacity. These results have implications for the clinical use of MBV as well as for the design of novel pUL97 kinase inhibitors.

## 1. Introduction

Infections with cytomegalovirus (CMV) can increase the risk of graft loss and mortality in transplanted patients [1]. The first class of antiviral agents ((val)ganciclovir, foscarnet and cidofovir) indicated for the prevention and treatment of CMV infections are inhibitors of the viral DNA polymerase [2]. More recently, additional classes of antiviral drugs that target other viral proteins such as the terminase complex (i.e., letermovir) and the pUL97 kinase (i.e., maribavir; MBV) were also approved for these indications [3]. Cyclopropavir (CPV; also known as filociclovir), a dual inhibitor of pUL97 kinase and viral DNA polymerase [4,5], is under development. The pUL97 serine/threonine protein kinase, encoded by the *UL97* gene, is a viral cyclin-dependent kinase (CDK) ortholog that shares structural and functional similarities with host CDKs. This enzyme is present as a tegument protein into virions [6] and is also expressed as an early–late gene product [7]. In the early steps of the viral replicative cycle, the protein is translocated to the nucleus through the binding of its nuclear localization signals (NLSs) to importin-α [8,9]. At later stages of infection, pUL97 can be detected in cytoplasmic compartments [10], containing reorganized cellular membranes where tegument formation and envelopment processes occur. The protein can be found in three isoforms due to alternative in-frame ATG initiation at residues M1, M74 and M157 [11]. The full-length protein (M1) is the predominant isoform in infected cells.

The activity of pUL97 kinase is critical for the regulation of CMV replication. This enzyme phosphorylates itself [12,13], as well as a series of viral and cellular proteins [14] involved in viral gene transcription/translation, viral DNA synthesis, cell cycle control, the nuclear egress of virions, and intrinsic immunity [15].

Viral pUL97 demonstrates partially overlapping functions with host CDKs. Cellular CDKs play an important role in the progression of the cell cycle by phosphorylating target proteins that control the transition from one step to the next. Among these, the retinoblastoma family of tumor suppressors (rb, p107 and p130) forms transcriptionally repressive complexes that arrest the progression of the cell cycle. During the cell cycle, the activity of cellular CDKs is dependent on cyclic variations in intracellular cyclin concentrations. Cyclins contain a domain, called a cyclin box (formed by several α-helices), that serves as an interface for binding to cellular CDKs [16]. The binding of cyclins to cellular CDKs induces changes in the active site conformation, whereas complete enzyme activation requires additional regulatory steps of site-specific phosphorylation. Like cellular CDKs, the viral pUL97 kinase phosphorylates transcriptional regulators in the rb protein pathway throughout the cell cycle, leading to the inactivation of rb proteins [17,18,19,20]. This results in an early cell-cycle S phase arrest associated with the dysregulation of cyclin levels and cellular CDK activity, called pseudomitosis [21]. pUL97 has been shown to interact with human cyclins B1, T1 and H [22,23,24], which may affect substrate recognition, self-interaction, autophosphorylation, and/or the recruitment of cellular CDKs to form ternary and higher-order complexes [25,26]. Furthermore, pUL97 kinase phosphorylates eukaryotic elongation factor 1 delta [27] and RNA polymerase II (initiation of immediate early gene transcription) [28], which are thought to promote viral gene expression.

During nuclear egress, it is proposed that nucleocapsids interact with the nuclear egress complex (NEC) formed by pUL50 and pUL53, and transit through the perinuclear space via envelopment at the inner nuclear membrane and subsequent budding at the outer nuclear membrane. The NEC then recruits pUL97 to the nuclear rim. pUL97 mimics cellular CDK1 activity during mitosis as it phosphorylates the nuclear lamina component, lamin A/C [29,30]. This leads to the disruption of the nuclear lamina [31], allowing the viral nucleocapsids to have access to the inner nuclear membrane for subsequent budding.

The pUL97 kinase does not appear to phosphorylate natural deoxynucleosides but it is implicated in the first phosphorylation step of GCV [32,33]. GCV monophosphate is then converted by cellular kinases into GCV triphosphate, which inhibits the viral pUL54 DNA polymerase activity [34,35]. In contrast, MBV is a selective inhibitor of pUL97 kinase activity [36]. Given the functional roles of pUL97 kinase in the CMV replication cycle, MBV inhibits viral DNA synthesis [36] and interferes with the morphogenesis and nuclear egress of nascent viral particles in infected cells [37]. Drug inhibition studies showed that MBV competes with ATP for binding to pUL97 kinase with an inhibition constant (K_i_) of 10 nM [38]. It was thus proposed that MBV is an ATP binding site inhibitor. As MBV may interfere with the phosphorylation of GCV, the co-administration of both drugs is expected to result in antagonistic effects against CMV infection [39]. MBV was also shown to be a competitive inhibitor of CPV phosphorylation by pUL97 kinase, with a K_i_ value of 3 nM [40]. This led to an apparently contradictory conclusion that MBV is a substrate phosphorylation site inhibitor. In 2021, MBV was approved by the Food and Drug Administration for the treatment of refractory/resistant CMV infections in adult and pediatric transplant recipients [41].

At the early stages of drug development, the first amino acid substitutions, conferring resistance to MBV, that were described in the literature were distinct from those induced by GCV. This seemed consistent with the fact that MBV is a specific inhibitor of pUL97 kinase activity, whereas GCV is a substrate of the enzyme. With time, the emergence of amino acid substitutions conferring cross-resistance to MBV and GCV has also been reported. This has important clinical implications as MBV is approved for the treatment of CMV infections that have not responded to (val)ganciclovir, foscarnet, or cidofovir. The first-line therapy against CMV infections is (val)ganciclovir and clinicians should be aware that some amino acid substitutions can confer cross-resistance to MBV and GCV. As the three-dimensional structure of pUL97 kinase has not been established by X-ray crystallography or cryo-electron microscopy yet, the role of amino acid substitutions in MBV resistance was analyzed using homology models.

More recently, artificial intelligence (AI) tools (such as I-TASSER [42], RoseTTAFold [43], AlphaFold2 [44], ColabFold (AlphaFold2_mmseqs2) [45], AlphaFold3 [46]) were developed to predict the three-dimensional structure of a protein from its amino acid sequence. Among these, AlphaFold2 is based on an artificial neural network with multiple layers of nodes, referred to as a deep learning algorithm [44]. AlphaFold2 includes an Evoformer module, which processes multiple sequence alignments (MSAs) to extract evolutionary information, and attention mechanisms, which process spatial interactions and evolutionary relationships to produce highly accurate predictions of three-dimensional protein structures [44]. The use of AlphaFold2 was validated in the 14th edition of the Critical Assessment of Structure Predictions in 2020 [44]. In this paper, we used AlphaFold2 to predict pUL97 kinase structure. The predicted protein structure was then used to examine the molecular mechanisms involved in resistance to MBV and cross-resistance to GCV.

## 2. Materials and Methods

### 2.1. Prediction of pUL97 Protein Structure

The three-dimensional structure of pUL97 protein was determined by the use of AlphaFold2. AlphaFold2 is an AI system developed by Google DeepMind (London, UK) that predicts a protein’s three-dimensional structure from its amino acid sequence [44,47]. To this end, the AI system exploits the information about related proteins, identified by MSAs. The pUL97 amino acid sequence from human CMV strain AD169 (GeneBank accession no. X17403) was used as a target. The full-length protein is composed of 707 amino acids (a.a.). The parameters used to run AlphaFold2 were as follows: use multimer model for monomers—yes; run relax—yes; relax using GPU—no; multimer model max num recycles—3. The MSA search found 58 unique sequences in 3 different databases (41 in UniRef90, 22 in small_BFD and 2 in MGnify). The MSA depth analysis was based on the normalized number of effective sequences for each residue (Figure S1).

### 2.2. Quality Assessment of Predicted pUL97 Protein Structure

**Figure 1:**
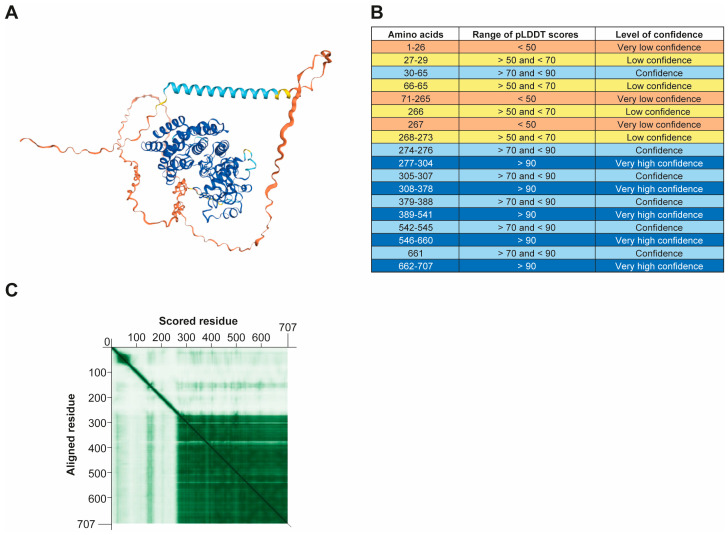
Predicted pUL97 protein structure and quality assessment. Structural representation of pUL97 protein structure predicted with AlphaFold2 colored according to different ranges of predicted local distance difference test (pLDDT) scores (**A**). Color code for ranges of pLDDT scores is shown in table (**B**). Predicted aligned error (PAE) plot for pUL97 protein structure (**C**). PAE plot shows scores of residue x with respect to aligned residue y for all pairs of residues in the protein. Dark green color indicates low PAE score, which corresponds to high reliability of relative position of residue, whereas light green color corresponds to lower confidence.

The quality of the pUL97 protein structure was estimated based on a predicted local distance difference test (pLDDT) and a predicted aligned error (PAE) plot provided by AlphaFold2 (both data files are available in Supplementary Materials). The pLDDT score estimates the confidence that the Cα atom (linked to COOH functional group) of the residue is correctly located relative to nearby residues [48]. The predicted pUL97 protein structure, generated with AlphaFold2, was colored according to the different ranges of pLDDT scores using Mol* 3D viewer [49] (Figure 1A). The color code for the ranges of pLDDT scores estimated for pUL97 three-dimensional structure is shown in Figure 1B. Regions predicted with very high confidence, confidence, low confidence, and very low confidence represent 58.4%, 8.1%, 2.1%, and 31.4% of the protein, respectively. Residues predicted with confidence (pLDDT > 70) range from a.a. 274 to 707 and cover 64.5% of the protein. The kinase domain of pUL97 (a.a. 337–651) was predicted with very high confidence (pLDDT  >  90), except for a.a. 379–388 and a.a. 542–545, which had confident predictive values (pLDDT > 70). Amino acids 383–388 and 542–545 had pLDDT values  >  85. However, residues 379–382 had lower pLDDT values (i.e., 81 for G379; 70 for the invariant glutamate E380; 70 for Q381; and 78 for Q382). Residues 1–273, which are predicted with a low confidence (pLDDT < 50), can potentially form disordered protein regions. The PAE scores estimate the positional error (in Å) of residue x when residue y is aligned in the predicted protein structure. This is represented as a heatmap. A PAE plot was drawn with a PAE viewer [50] and it showed a level of high confidence (depicted by dark green color) in the relative position of each residue compared to aligned residues in the protein structure for the a.a. 274 to 707 region of pUL97, whereas the confidence was low (shown by light green color) in the potentially disordered region (Figure 1C).

The assessment of model quality at both the global and local levels was further evaluated using MolProbity version 4.5.2 [51,52]. Reduce software was run within MolProbity to add and optimize missing hydrogens. The clash score and MolProbity score for the predicted pUL97 protein structure were estimated at 0.92 (rank in 99th percentile) and 1.79 (rank in 86th percentile), respectively.

We also attempted to predict the structure of pUL97 kinase protein using other AI systems, such as ColabFold v1.5.5 [45] and I-TASSER (Iterative Threading ASSEmbly Refinement) [42]. However, based on pLDDT values, the reliability of the pUL97 protein structure, predicted with both AI tools, was lower than that obtained with AlphaFold2. Higher clash scores and Molprobity scores were also obtained for the models generated with ColabFold (26.05 (20th percentile) and 3.16 (17th percentile), respectively) and I-TASSER (8.49 (79th percentile) and 3.23 (15th percentile), respectively). Thus, AlphaFold2 provided a better prediction of the three-dimensional structure of pUL97 kinase compared to ColabFold and I-TASSER.

### 2.3. Docking of ATP and Antiviral Drugs to the Predicted pUL97 Protein Structure

The docking of ATP, MBV, GCV, and CPV molecules to the predicted pUL97 protein structure was performed by using grid-based ligand docking with energetics (GLIDE) in Maestro software version 14.3.129 (Schrödinger, LLC, New York, NY, USA) [53]. The complete predicted pUL97 protein structure was used for docking calculation. The receptor grid was defined to include the residues of the invariant lysine (K355) and the catalytic aspartic acid (D456). The pUL97 protein kinase was prepared using the Maestro software version 14.3.129 to resolve missing hydrogen atoms, ambiguous protonation states, and flipped residues. LigPrep in Maestro software version 14.3.129 was used to convert the different ligand files (in SD format) into single, low-energy, three-dimensional structures with correct chiralities. After docking calculation, Maestro software version 14.3.129 provides a ligand interaction diagram that shows the hydrogen bonds, salt bridges, and hydrophobic contacts between the ligand and the predicted pUL97 protein. PyMOL software version 3.1.3 (Schrödinger, LLC) [54] was used to determine the distance between selected atoms of the ligands and the predicted pUL97 protein structure.

### 2.4. Generation of Mutant pUL97 Homology Models

All mutant pUL97 protein structures harboring amino acid substitutions that conferred resistance to MBV were generated by homology modeling using the Swiss Model (Computational Structural Biology Group, Basel, Switzerland) [55]. The wild-type pUL97 protein structure predicted with AlphaFold2 was used as a template. Missing hydrogens were then added and optimized in all mutant protein models using the Reduce software within MolProbity version 4.5.2 [51,52]. The quality assessment of mutant proteins models was performed based on the root mean square deviation (RMSD), the MolProbity score, and clash score (Table S1). RMSD is used to estimate the similarity between two protein structures. The RMSD values of mutant proteins generated with the Swiss Model software, superimposed onto the wild-type pUL97 protein predicted with AlphaFold2, were calculated using PyMOL version 3.1.3 [54]. All mutant protein models had RMSD values with Cα coordinates of less than 0.2 Å for 707 residues when compared with the predicted wild-type protein structure, with the lowest value being obtained for the H411Y mutant (0.122 Å). The MolProbity and clash scores of the mutant protein models were estimated with MolProbity version 4.5.2 [51,52]. MolProbity scores range from 1.61 (C151G mutant) to 1.85 (P521L mutant). Clash scores range from 0.55 (C151G mutant) to 1.20 (P521L mutant). Predicted wild-type and mutant pUL97 protein structures were drawn and examined using PyMOL molecular visualization software version 3.1.3 [54].

## 3. Results and Discussion

### 3.1. Conserved Structural Sub-Domains and Functional Domains of pUL97 Kinase

**Figure 2:**
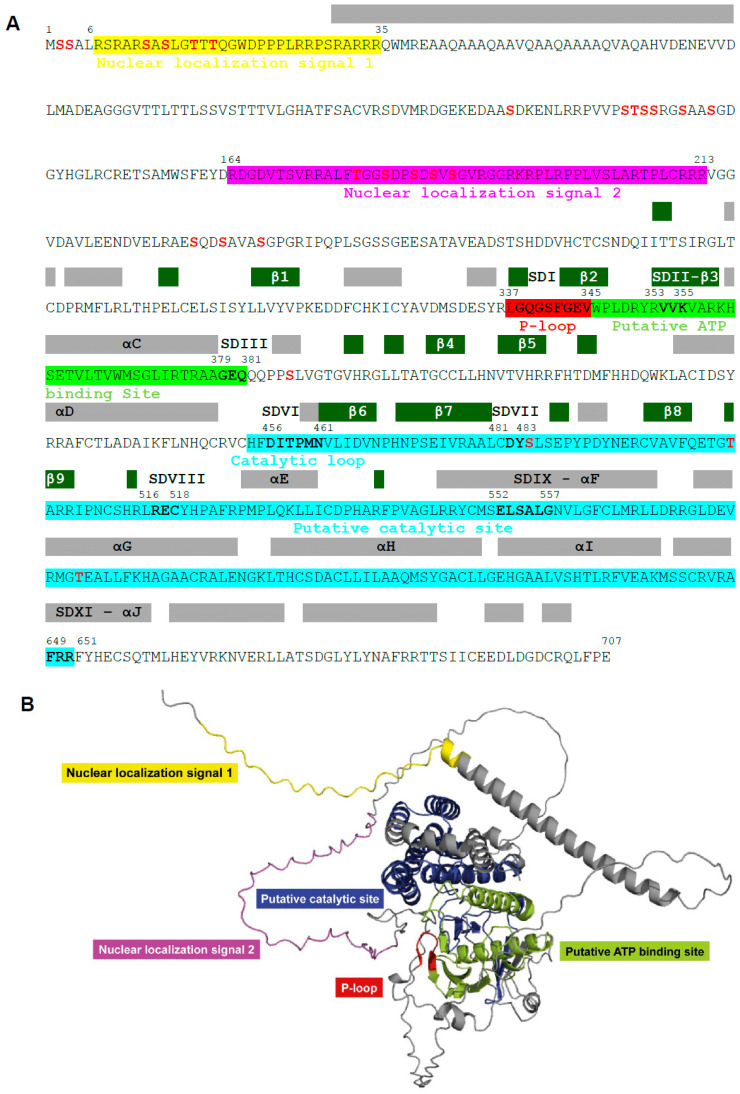
Conserved structural sub-domains and functional domains of pUL97 kinase. Amino acid sequence of pUL97 kinase of human cytomegalovirus strain AD169 annotated with conserved structural sub-domains (SD) among cellular and viral protein kinases and functional domains [56,57,58] that include nuclear localization signal 1 (in yellow), nuclear localization signal 2 (in magenta), putative ATP binding site (in green), P-loop (in red), and putative catalytic site (in blue) (**A**). α-helices (α) and β-strands (β) in predicted pUL97 protein are shown with gray and green boxes, respectively. These secondary structures are labeled according to convention for cAMP-dependent protein kinase. Phosphosites (as reviewed in [25]) are also indicated in bold red. Structural representation of functional domains on predicted pUL97 kinase structure (**B**).

Conserved structural sub-domains and functional domains, established by sequence homology between cellular and viral protein kinases [56,57,58], are shown on the a.a. sequence of pUL97 kinase of human CMV strain AD169 in Figure 2A. The amino–terminal part of the predicted pUL97 protein, which contains the NLS1 and NLS2 (a.a. 6–35 and a.a 164–213, respectively) [8,9], is poorly structured (Figure 2B). The carboxy–terminal part, which corresponds to the kinase domain (a.a. 337–651), displays a globular structure [59]. Like other protein kinases, the predicted pUL97 core is folded into two lobes interconnected by a hinge region. The N-lobe is primarily composed of 5 β-strands and also includes the αC-helix, whereas the larger C-lobe is mainly formed by α-helices [60]. The N-terminal lobe, the hinge region, and the C-terminal lobe form a deep hydrophobic cleft that corresponds to the ATP binding site. The substrate binding site is a shallow, open surface in the C-terminal lobe that facilitates protein–protein interactions. Amino acids involved in the formation of α-helices and β-strands (as determined with the Swiss Model [55]) are shown on the a.a sequence of pUL97 kinase (Figure 2A). These secondary structures are labeled according to the convention established for the cyclic AMP-dependent protein kinase (cAPK).

Protein kinases of herpesviruses contain most of the eleven conserved structural sub-domains of protein kinases, with the exception of sub-domains IV, V and X [56,57,58]. The putative catalytic site (a.a. 456–651) of the enzyme is located in sub-domain VI and includes the catalytic loop (a.a. 454–461) with the catalytic aspartic acid residue at position 456. The putative ATP binding site (a.a. 337–453) is located in sub-domains I to III. Sub-domain I corresponds to the P-loop (a.a. 337–345), which contains the glycine-rich motif (GXGXXG). Sub-domain II contains an invariant lysine residue at position 355, which is essential for kinase activity. Indeed, the complete loss of kinase activity was observed after deletion (i.e., K355del) or substitutions (i.e., K355M or K355Q) of this amino acid [12,61]. The invariant K355 is involved in the correct configuration of the phosphate groups of ATP, so that the γ-phosphate is in line with the substrate for phosphoryl transfer [60]. Several serine and threonine phosphorylation sites were identified in pUL97 kinase, as reviewed in [25], and they are shown in Figure 2A.

### 3.2. Functional Differences Between Cellular Protein Kinases and Viral pUL97 Kinase

Despite the fact that functional residues in the ATP binding site and catalytic center are conserved between CDKs and pUL97 kinase [56,57,58], several structural and functional differences exist between them. For instance, CDK2 contains a PSTAIRE-like cyclin-binding domain (in the αC-helix) and an activation segment (in the T-loop), which are involved in the process of the activation of the enzyme [62]. In CDK2 apoenzyme, the invariant lysine (K33) binds to the α- and γ-phosphate oxygen groups of ATP and the molecule is not correctly oriented for catalysis. Furthermore, access to the substrate binding site is restricted by the T-loop [62]. The kinase is partially activated by the binding of cyclin A, which induces the rotation of the activation segment [63,64] and promotes the formation of a salt bridge between the main chain carbonyl of the glutamate (E51) residue in the αC-helix and the invariant K33. The K33 residue can then bind to the α- and β-phosphate oxygen groups of ATP, which align the γ-phosphate with the catalytic aspartic acid (D145) residue for the phosphoryl transfer reaction [63]. This conformational change also causes the displacement of the T-loop that relieves the blockade of the catalytic cleft [63,64], a crucial step for the recognition of substrates [65]. The full kinase activity is then achieved by phosphorylation of the exposed threonine (T160) in the T-loop using CDK-activating kinase [63,65]. This step does not markedly affect the substrate binding affinity but increases the rate of phosphoryl transfer reaction [66].

In contrast to CDKs, the viral pUL97 kinase lacks the PSTAIRE-like cyclin binding domain. Nevertheless, the pUL97 kinase has been shown to interact with human cyclins B1 (at a.a. 231–280 and 361–532), T1 (at a.a. 363–647), and H (at a.a. 328–532) with different affinities [22,23,24]. The binding domain of cyclin T1 overlapped with the pUL97 self-interaction domain (a.a. 231–280) and it was shown that cyclins T1 and H may play a bridging function in pUL97-pUL97 dimerization and oligomerization processes, which are associated with autophosphorylation/autoregulation [22,26]. However, the autophosphorylation of pUL97 kinase is not required for exogenous substrate phosphorylation [13]. Furthermore, no interaction could be observed between cyclin B1 and pUL97 kinase inactivated by mutagenesis or inhibited by MBV, suggesting that cyclins can only bind to an active enzyme [23]. In enzymatic assays, the activity of pUL97 kinase produced with a baculovirus expression system (in the absence of cyclins) is potently inhibited by MBV [38,40]. Taken together, these data suggest that the active site of pUL97 kinase may constitutively adopt a functional conformation without the need for cyclin binding.

In the absence of a crystal structure of pUL97 kinase, the role of amino acid substitutions conferring MBV resistance was predicted using homology models. These models were constructed based on the structure of several crystallized proteins, such as yeast general control kinase 2 (GCN2; PDB 1zyc), a mutant GCN2 complexed to ATP (PDB 1zyd) [67,68], human serine–arginine protein kinase 1 (SRPK1; PDB 1wbp) [67], human CDK2 (PDB 1di8) [69], and human CDK2 complexed with ATP (PDB 1hck) [70]. However, sequence identity between the viral pUL97 kinase and cellular kinases ranges from 15% to 20%, which limits the accuracy of these homology models. More importantly, all these homology models were generated based on the structures of protein kinases in their inactive state. Indeed, the yeast wild-type GCN2 kinase structure (PDB 1zyc) was crystallized in its apostate, which restricts ATP binding, whereas the active form of the enzyme, binding and hydrolyzing ATP, was obtained by mutagenesis (PDB 1zyd) [71]. The human CDK2, without ATP (PDB 1di8) or complexed with ATP (PDB 1hck), was also not crystallized in an activated state [72,73]. Therefore, the active site of these protein models did not adopt a conformation where the phosphate groups of ATP was correctly oriented for catalysis and the substrate phosphorylation site was accessible.

In contrast to CDKs, the activation of most protein kinases requires site-specific phosphorylation in the activation loop [60]. These protein kinases possess a conserved HRD motif that contains the catalytic aspartic acid residue. The positively charged arginine in the HRD motif correctly orients the activation loop [74] to configure the phosphorylation site, the catalytic loop, and the magnesium-binding loop for catalysis. The pUL97 kinase lacks the arginine in the HRD motif (which is rather 454-HFD-456). Kinases lacking this arginine are not phosphorylated in the activation loop [60]. The threonine (T195) in the activation loop of cAPK is constitutively phosphorylated and the enzyme is active by itself [75]. The E91 located in the αC-helix thus interacts with the invariant K72 without the need for an activation step [76]. In the predicted pUL97 kinase structure, the E362 residue in the αC-helix is located at 2.7 Å of K355 (Figure S2), which should be sufficient to establish an interaction. This interaction is required for the proper positioning of the phosphate groups of ATP for catalysis. In most inactive protein kinases, the phenylalanine of the conserved DFG motif is out (DFG-out) and moves in a hydrophobic pocket between the N-lobe and the C-lobe (DFG-in) for enzyme activation [77]. This leads to the proper positioning of the aspartic acid residue of the DFG motif that is involved in the coordination of Mg^2+^ ion(s) in the catalytic site. The pUL97 kinase has no DFG motif and the aspartic acid residue at position 481 might be correctly positioned in the catalytic site for the coordination of Mg^2+^ ions (Figure S2). We may thus assume that the active site of the pUL97 kinase structure predicted with AlphaFold2 should adopt a functional conformation that may allow the correct orientation of the phosphate groups of ATP for catalysis and access to the substrate phosphorylation site.

### 3.3. Docking of ATP and Antiviral Drugs to Predicted pUL97 Protein Structure

**Figure 3:**
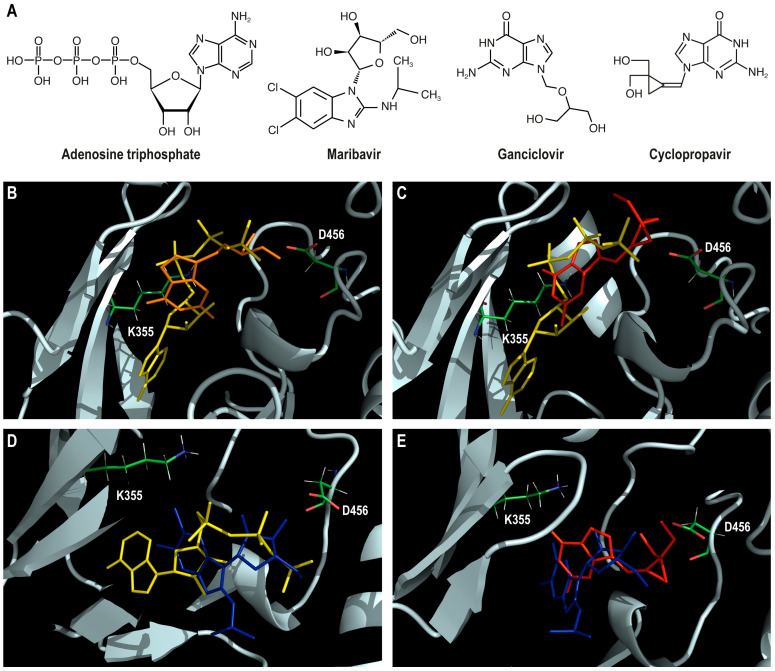
Chemical structures of adenosine triphosphate (ATP), maribavir (1-H-β-L-ribofuranoside-2-isopropylamino-5,6-dichlorobenzimidazole), ganciclovir (9-[1,3-dihydroxy-2-propoxymethyl] guanine), and cyclopropavir ((Z)-9-((2,2-bis-(hydroxymethyl)cyclopropyldiene)methyl)guanine) (**A**). Selected views of most probable poses of ATP with ganciclovir (**B**), ATP with cyclopropavir (**C**), maribavir with ATP (**D**), and maribavir with cyclopropavir (**E**), docked separately and then superimposed onto predicted pUL97 protein structure (in pale cyan) to show absence (**B**,**C**) and presence (**D**,**E**) of clash between the different pairs of ligands. ATP, maribavir, ganciclovir, and cyclopropavir are shown using yellow, blue, orange, and red sticks, respectively. Catalytic D456 and invariant K355 residues are also shown using green sticks.

The structure of the pUL97 protein, predicted with AlphaFold2, was used for the docking of ATP and antiviral drugs. The chemical structures of ATP, MBV, GCV, and CPV are shown in Figure 3A. Flexible ligand docking with Maestro software version 14.3.129 generated a series of poses for the predicted protein structure. In order to perform matching with structural and biochemical data, we established 4 assumptions to select the best poses of ATP and antiviral drugs. These 4 assumptions were as follows: (a) the α- and β-phosphate oxygen groups of ATP should interact with the K355 residue and the γ-phosphate should be positioned in proximity to the catalytic D456; (b) GCV and CPV, which are substrates of pUL97 kinase, should not clash with ATP; (c) the hydroxyl groups of GCV and CPV, which are phosphorylated by pUL97 kinase, should interact with the catalytic D456; (d) MBV, which is a competitive inhibitor of ATP binding and CPV phosphorylation, should clash with both molecules. The projected positions of ATP with GCV molecules, ATP with CPV molecules, MBV with ATP molecules, and MBV with CPV molecules, posed separately and then superimposed onto the predicted pUL97 protein structure, are shown in Figure 3B–E, respectively. As expected, the most favorable poses of ATP and antiviral drugs were located in the cleft between the N-lobe and the C-lobe of the predicted pUL97 kinase (Figure S3).

Diagrams of interactions of ATP and antiviral drugs with the predicted pUL97 kinase, generated by Maestro software version 14.3.129, are shown in Figure S4. The docking scores of ATP, GCV, CPV, and MBV were −4.6, −4.7, −4.9, and −4.9 Kcal/mol, respectively.

#### 3.3.1. Docking of ATP to pUL97 Kinase

The adenine group of ATP is located in a hydrophobic pocket consisting of F342 (pLDDT = 92), V353 (pLDDT = 97), V345 (pLDDT = 97), L397 (pLDDT = 97), R412 (pLDDT = 96), R413 (pLDDT = 96), and F414 (pLDDT = 96) residues. The ribose group may interact with N461 (pLDDT = 97) via hydrogen bonding. As expected, our docking results showed that the invariant K355 (pLDDT = 97), which may interact with E362 (pLDDT = 96) in the αC-helix, can establish a salt bridge with the α-phosphate oxygen of ATP located at 4.1 Å (Figure S4A). However, no interaction was seen between K355 and the β-phosphate oxygen group, which was located at 5.7 Å. This discrepancy may be due to the high flexibility of the phosphate moiety of ATP, as well as the lack of metal ions and water molecules in our system. Residues of the glycine-rich loop (337-LGQGSFGEV-345) may also help to align the phosphate moiety of ATP for phosphoryl transfer. The α-phosphate oxygen group may interact with F342 (pLDDT = 92) and G343 (pLDDT = 94) via hydrogen bonds. The β-phosphate of ATP oxygen group may establish hydrogen bonding with S341 (pLDDT = 91). The γ-phosphate oxygen group of ATP may establish hydrogen bonding with T458 (pLDDT = 97).

In inactive CDK2 and cAPK, the configuration of the α- and γ-phosphates of ATP is almost similar, but the β-phosphate is oriented on opposite sides [62]. As a result, the number and coordination of Mg^2+^ ion are different for the two enzymes. In inactive CDK2, there is only one Mg^2+^ ion, which is coordinated by the three phosphate oxygen groups of ATP [62]. When CDK2 is in its active state (when E51 in the αC-helix interacts with the invariant K33), the coordination of the Mg^2+^ ion is achieved by the α- and γ-phosphate oxygen groups of ATP and the catalytic D145 residue [63]. In contrast, in cAPK, there are two Mg^2+^ ions. The activating high-affinity Mg^2+^ ion is coordinated by the β- and γ-phosphates and an aspartic acid (D184), residue whereas the inhibitory low-affinity Mg^2+^ ion is coordinated by the α- and γ-phosphates of ATP and by asparagine (N171) and the D184 residues [78].

In the predicted pUL97 kinase, the orientation of the β-phosphate is closer to that in cAPK. The residues D481 and N461 are positioned on each side of the phosphate moiety of ATP (Figure S2) and might be involved in the coordination of two Mg^2+^ ions. The invariant K335 may interact with D481 residue as they are separated by 2.8 Å. Furthermore, the amide group of N461 is at hydrogen bonding distance from the carboxyl group of the catalytic D456 (3.1 Å), which may stabilize the catalytic loop, as reported in cAPK [78]. T458 may also stabilize the γ-phosphate of ATP through hydrogen bonding in the same way as lysine (K168) residue in cAPK. We may thus assume that the coordination of Mg^2+^ ions in the predicted pUL97 kinase might be closer to cAPK than to CDK2. AlphaFold3 can predict the structure of a protein and its interaction with some ligands such as ATP and Mg^2+^ [46]. The prediction of the structure of the pUL97 kinase with ATP and Mg^2+^ ions using AlphaFold3 showed that two Mg^2+^ ions could be accommodated in the active site of the enzyme (Figure S5). A Mg^2+^ ion may be coordinated by the β- and γ-phosphate oxygen groups of ATP and the D481 residue, whereas the other Mg^2+^ ion may be coordinated by the α- and γ-phosphate groups and by the N461 and D481 residues. Homology models based on CDK2 may be thus not appropriate for extrapolating the binding of ATP to pUL97 kinase.

#### 3.3.2. Docking of GCV and CPV to pUL97 Kinase

As GCV is a substrate of pUL97 kinase, its projected position does not clash with that of ATP in the predicted protein structure (Figure 3B). The guanine group of GCV is almost perpendicular to the ribose group of ATP and the dihydroxy-2-propoxymethyl side chain of GCV is located between the β- and γ-phosphate groups. The guanine group of GCV may establish hydrogen bonding with N461 (Figure S4B). The dihydroxy-2-propoxymethyl side chain may interact with T458 and D456 (pLDDT = 96) by hydrogen bonding. The distances between the two hydroxyl groups of GCV and the carboxyl group of the catalytic D456 residue were estimated to be 2.6 Å and 3.2 Å, respectively. During the phosphoryl transfer reaction, the catalytic aspartic acid deprotonates the hydroxyl group of the substrate and catalyzes the nucleophilic attack using the hydroxyl oxygen on the γ-phosphate. This leads to an inversion of the phosphorus configuration that requires that the γ-phosphate of ATP to be positioned in line with the hydroxyl group of the substrate to allow for phosphoryl transfer. The distances between the γ-phosphate of ATP and the two hydroxyl groups of GCV were, respectively, estimated to be 3.0 Å and 3.8 Å, which may be sufficient to allow drug phosphorylation. Based on the crystal structure of cAPK, its was estimated that the distance between the hydroxyl group of a peptide substrate and the carboxyl group of the catalytic D166 residue was 2.8 Å, whereas it was 2.7 Å with the γ-phosphate of ATP [78]. Therefore, one of the two hydroxyl groups of GCV may be phosphorylated by pUL97 kinase to produce GCV monophosphate.

The projected position of CPV in the predicted pUL97 kinase does not clash with that of ATP, except for the NH_2_ in the guanine moiety that touches the ribose of ATP (Figure 3C). CPV is both a substrate and an inhibitor of pUL97 kinase [4,5]. The dual mode of action of CPV might be related to the sequential order of ATP and drug binding. In such a way, pUL97 kinase bound with ATP might phosphorylate CPV, whereas CPV bound to the enzyme might inhibit its activity by preventing ATP binding. The CPV molecule is almost parallel to the phosphate moiety of ATP, except for the two hydroxymethyl groups of CPV, which are located on each side of the γ-phosphate. The guanine group may interact with M460 (pLDDT = 95) and S341 by hydrogen bonding (Figure S4C). One hydroxyl group may establish a hydrogen bond with D456. The distance between the closest hydroxyl group of CPV and the catalytic D456 residue was estimated to be 3.0 Å, and it was separated from the γ-phosphate of ATP by 3.8 Å. We suggest that, due to proximity and the steric hindrance caused by the cyclopropane moiety, only one hydroxyl group of CPV could be phosphorylated. This observation is in line with the stereoselective phosphorylation of CPV by pUL97 kinase, which leads to the formation of the (+)-enantiomer of CPV monophosphate [40].

#### 3.3.3. Docking of MBV to pUL97 Kinase

Homology models based on yeast GCN2 kinase (PDB 1zyc), human SRPK1 (PDB 1wbp), and human CDK2 (PDB 1di8) structures were used to deduce the binding site of MBV to pUL97 kinase. Residues V353, T409, H411, and L397, which may confer resistance to MBV once substituted, were used to guide drug docking. The projected position of the MBV molecule was located between residues V353 and T409 to H411 in a cleft between the P-loop and the catalytic loop [67,69]. The position of the benzimidazole ring of MBV was also predicted to overlap with the adenine group of ATP. From these homology models, it was thus suggested that MBV was a competitive inhibitor of ATP binding [38]. However, such an overlap may be not consistent with the observation that MBV is also a competitive inhibitor of CPV as the antiviral drug is mainly localized in the putative ATP binding site and not so much in the substrate phosphorylation site.

By using a more refined homology model based on 6 templates selected by heuristics, where ATP and Mg^2+^ were imported from a kinase-associated phosphatase in complex with phosphoCDK2 (in an activated conformation; PDB 1fq1), another group suggested that MBV may establish interactions with H147, C480, D481, T458, R412, and F414 [79]. Furthermore, the ribose group of MBV and the catalytic D456 residue were involved in the coordination of a Mg^2+^ ion. This can be due to the fact that, in active CDK2, the catalytic D145 coordinates the Mg^2+^ ion, together with the α- and γ-phosphate oxygen groups of ATP [63]. Our prediction of pUL97 kinase with ATP and Mg^2+^ using AlphaFold3 suggests that the active site of the enzyme might accommodate two Mg^2+^ ions. The configuration of ATP and metal ions in active CDK2 may not be appropriate to be superimposed to homology models of pUL97 kinase.

A previous study reported that the kinase domain of pUL97, predicted with AlphaFold, was used to dock MBV [80]. Results showed that the ribose group of MBV may establish hydrogen bonds with G343, K355, and D481 residues. It was suggested that the drug is located in the ATP binding pocket, as reported by others [67,69,79]. However, the version of AlphaFold used, the quality assessment of the predicted protein, and the method of selecting the best pose of MBV to the protein were not detailed.

**Figure 4:**
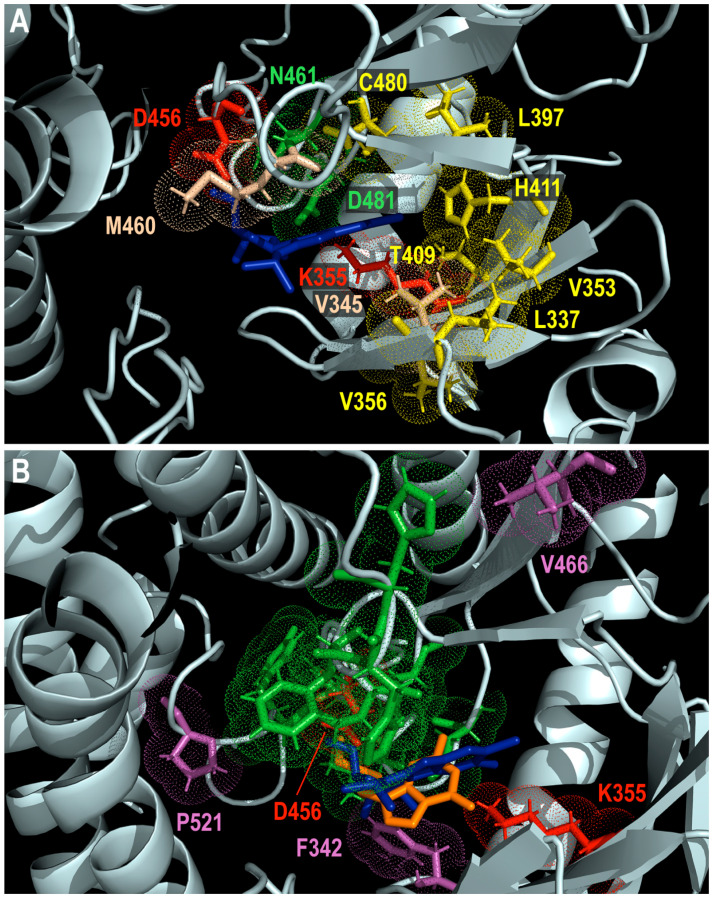
Distribution of amino acids that are substituted to confer resistance (yellow sticks) or hypersusceptibility (wheat-colored sticks) to maribavir (**A**) or cross-resistance to maribavir and ganciclovir (magenta sticks; (**B**)) in predicted pUL97 protein structure (pale cyan). Invariant K355 and catalytic D456 residues are represented with red sticks. Residues represented by green sticks may be indirectly involved in drug resistance and their implication is discussed with each specific amino acid substitution in text. Projected positions of maribavir (blue sticks) and ganciclovir (orange sticks) are also shown. Dots show atomic radius.

Using our model, predicted with AlphaFold2, we showed that the projected position of the benzimidazole ring of MBV may clash with the ribose group of ATP, whereas the ribofuranoside group may clash with the phosphate moiety of ATP (Figure 3D). Thus, this result supports the observation that MBV is an inhibitor of ATP binding to pUL97 kinase, as reported previously [38]. Furthermore, the benzimidazole ring of MBV may clash with the guanine group of CPV (Figure 3E) and with the guanine group of GCV (Figure S6). These observations suggest that MBV is also a substrate phosphorylation site inhibitor, as reported in [40]. The ribose group of MBV may establish hydrogen bonds with D481 (pLDDT = 96) and D456 (Figure S4D). However, the projected position of MBV is not located between residues V353 and T409 to H411, as suggested [67,69], but rather in a cleft formed by several residues (V356, V345, L337, V353, T409, H411, L397, and C480) that may confer resistance to MBV once substituted (Figure 4A). This discrepancy may be due to the fact that the relative positions of V353 and T409 to H411 in the homology models [67,69] are different compared to their locations in our model predicted with AlphaFold2. The close conformation of the active site (which is not configured for catalysis) of these homology models, based on inactive kinases, compared with the functional active site conformation of the protein structure, predicted with AlphaFold2 as used in our study, may be at the origin of this difference.

In our docking experiments, most residues of pUL97 kinase, generated with AlphaFold2, that may be involved in the binding of ATP and antiviral drugs were predicted with a very high degree of confidence (pLDDT > 90). In the kinase domain, only residues 379–382, which include the GEQ motif in the conserved structural sub-domain III located in the putative ATP binding site, were predicted with a lower degree of confidence (with pLDDT values between 70 and 78). As shown in Figure S7, these 4 residues are not located near the docking sites of ATP and antiviral drugs. Therefore, we may assume that the lower prediction for these residues did not interfere with the docking of ATP and antiviral drugs to the protein.

### 3.4. Insights into the Mechanisms of MBV-Induced Inhibition of pUL97 Kinase Activity

Two apparently contradictory hypotheses have been proposed to explain the mechanisms of the MBV-induced inhibition of the pUL97 kinase. Kinetics studies of pUL97 autophosphorylation in the presence of MBV showed that the drug is a competitive inhibitor of ATP binding [38]. The K_i_ value was estimated at 10 nM. As intracellular ATP concentrations are typically in the mM range and K_m_ values of most protein kinases for ATP are in the µM range (i.e., 27 µM for the pUL97 kinase [38]), the potencies of ATP binding inhibitors, determined in biochemical and cellular assays, are not generally in agreement [81]. Indeed, the effective concentrations of MBV determined by susceptibility testing, with results ranging from 0.1 µM to 13 µM [3], were higher than those reported in biochemical assays. Another study showed that MBV is also a competitive inhibitor of CPV phosphorylation, with a K_i_ value of 3 nM suggesting that the drug could be a substrate phosphorylation site inhibitor [40]. Our docking results suggest that both mechanisms could be involved and that MBV could be a dual inhibitor of ATP binding and substrate phosphorylation.

MBV also prevents downstream activities of pUL97, such as pp65 phosphorylation [82,83], the S2 phosphorylation of lamin A/C [29,84], and rb phosphorylation [11,18]. In contrast to CDK2, which uses an RxL motif to recognize rb [85], pUL97 kinase uses a LxCxE motif [17,18]. The LxCxE sequence belongs to short linear motifs (SLiMs), which are usually located in intrinsically disordered regions and are involved in protein binding [86]. The LxCxE motif binds to the conserved LxCxE binding pocket in the rb domain. This motif has been found in cellular proteins that interact with rb, as well as in viral proteins (such as the human papillomavirus E7 oncoprotein) that bind and suppress the function of rb family proteins [87]. In the predicted pUL97 kinase, the LRCRE residues are located at positions 149 to 153 in the structurally disordered region, which is directed to the cleft between the N-lobe and the C-lobe (Figure S8A). Several phosphosites (i.e., S133, T134, S135, S136, S139 and S142) were identified in this region of the protein [25]. Recombinant virus expressing the isoform M157 of pUL97 kinase showed markedly reduced viral growth and a high level of resistance to MBV while retaining susceptibility to GCV [11]. This suggests that this region (i.e., amino acids 1–157) is involved in functions critical in enzyme activity. C151G amino acid substitution in the LRCRE motif was shown to reduce rb phosphorylation [18] and to induce hypersusceptibility to MBV, but not to GCV [88]. The comparison of the G151 mutant protein with the wild-type pUL97 kinase revealed that the substitution of cysteine (Figure S8B) with glutamine (Figure S8C) at position 151 may disrupt the interaction with L149 and may increase the distance from F160. These two residues are part of the hydrophobic pocket surrounding the isopropylamino group of MBV (Figure S8B). These changes may thus affect MBV susceptibility, either by increasing drug binding or by another mechanism. These observations suggest that the mechanism of action of MBV may be related to the function of the Rb binding motif and that the drug might affect the phosphorylation of proteins that use the LRCRE motif to bind to the pUL97 kinase.

### 3.5. Amino Acid Substitutions Conferring Resistance to Maribavir

The ATP binding pocket of protein kinases is composed of a buried region and a solvent-accessible region, access to which is controlled by the DFG motif and the gatekeeper residue (that confers selectivity for nucleotide binding), respectively. Inhibitors that target the ATP-binding site can induce the rapid emergence of mutations at the gatekeeper residue and within the P-loop, the hinge region, the activation loop, and the C-lobe [89]. The mutation of the gatekeeper residue alters the size of the side chains, which sterically hinders the inhibitor access to the hydrophobic pocket at the back of the ATP binding site cleft, reducing drug binding and efficacy. These mutations induce drug resistance but they do not change (or they can increase) the enzyme activity and/or ATP binding affinity [90]. In contrast, substrate phosphorylation site inhibitors can induce the emergence of mutations that reduce both inhibitor binding and enzyme activity.

Table 1 shows the characteristics of recombinant viruses harboring amino acid substitutions (levels of drug resistance to MBV and/or GCV as well as viral growth) that were analyzed in this study.

The distribution of amino acid substitutions that confer resistance/hypersusceptibility to MBV (Figure 4A) or cross-resistance to MBV and GCV (Figure 4B) in the predicted pUL97 kinase is also represented relative to the projected positions of antiviral drugs.

All mutant pUL97 kinase structures were generated by homology modeling, using the predicted wild-type protein structure as a target. The impacts of amino acid changes on mutant pUL97 models were then compared to the wild-type protein structure. This analysis revealed that amino acid substitutions conferring resistance to MBV may comprise two main clusters. The first cluster may be associated with the region surrounding the invariant K355 (in the structural sub-domain II located in the putative ATP binding site) and the second cluster may be associated with residues D456 and N461 in the catalytic site (in the structural sub-domain VI).

The first cluster includes T409M, H411L/N/Y, and C480F/R substitutions that were detected in clinical specimens obtained from patients treated with MBV. In clinical trials of MBV, the T409M, H411Y, and C480F mutations represented 61%, 13%, and 26% in *UL97* genotyping follow-up for patients with CMV clearance and recurrences while on MBV [101]. The other amino acid changes were selected in vitro and included L397R (selected under 2916W93 compound (a carboxylic analog of MBV)), L337M, V353A (both selected under MBV), and V356G (selected under CPV).

**Figure 5:**
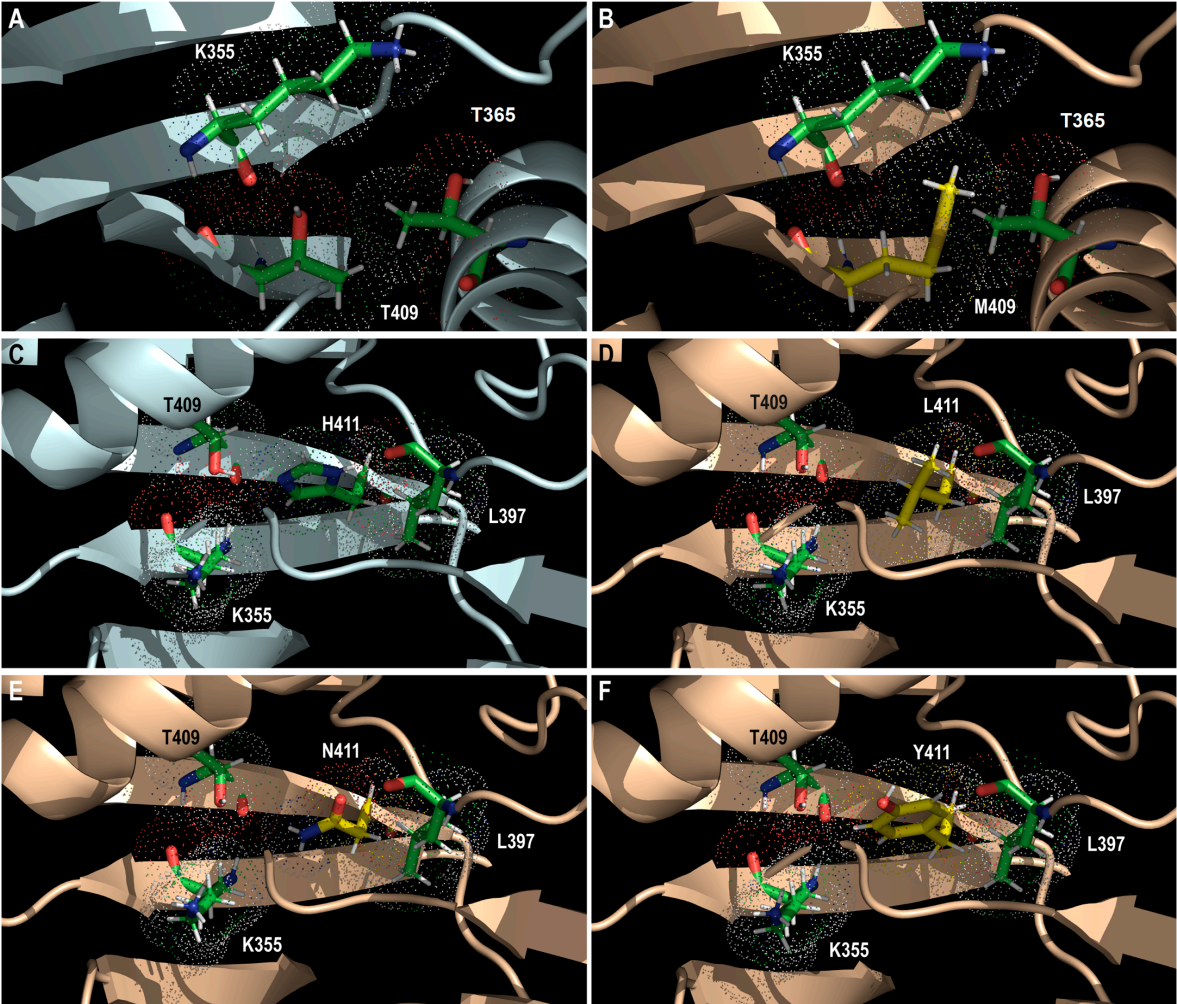
The structural representation of T409M and H411L/N/Y amino acid substitutions. The predicted wild-type (T409) pUL97 protein structure (in pale cyan; (**A**)) and a model of the M409 mutant (in wheat color; (**B**)). Wild-type T409 and mutated M409 residues are shown using green sticks and using yellow sticks, respectively. Residues K355 and T365 are also shown using green sticks. The predicted wild-type (H411) pUL97 protein structure (in pale cyan; (**C**)) and a model of L411 (**D**), N411 (**E**) and Y411 (**F**) mutants (in wheat color). The wild-type H411 residue is shown using green sticks, whereas mutated L411, N411, and Y411 residues are shown using yellow sticks. Residues L397, T409, and K355 are also shown using green sticks. Dots show the atomic radius.

T409M substitution confers high-grade resistance to MBV (81-fold increases in 50% effective concentration (EC_50_) of the wild type) [91]. The T409M mutant recombinant virus was as susceptible to GCV as the wild type. The growth of the recombinant T409M mutant virus was not affected compared to that of the wild type [91]. The substitution of threonine (Figure 5A) with bulky methionine (Figure 5B) at position 409 may alter the shape of the cavity around the invariant K355 and thus the docking of MBV. Furthermore, M409 may change its interactions with T365 located in the αC-helix (i.e., 2 hydrogen bonds compared to only 1 for T409). Protein kinase domain contains a hydrophobic regulatory spine (R-spine) that consists of 4 residues (RS1–RS4); this is assembled in the active conformation and disassembled in the inactive conformation [77]. The R-spine is one of the key dynamic elements that regulates the function of protein kinases. The R-spine links the two lobes of the protein and forms a flexible spine that coordinates their movements during catalysis [77]. Based on sequence homology with other protein kinases [102], RS1, RS2, and RS4 residues should correspond with H454 (in the 454-HFD-456 motif), Y482 (in the 481-DYS-483 motif), and possibly C402 (in the β4-strand) in the predicted pUL97 kinase, respectively [102]. RS3 residue should correspond with T365 as it is located 4 C-terminal residues from the αC-helix glutamate (E362 in pUL97), which may interact with the invariant lysine (K355 in pUL97) (Figure S9). Furthermore, a shell is formed by three residues (SH1-SH3) that are located close to RS3 and RS4. The access to the hydrophobic pocket of the ATP binding site (and towards RS3) is controlled by SH2, which is also called the gatekeeper residue. The gatekeeper is a residue of the β5-strand, which is located close to or interacts with RS3. The mutation of the gatekeeper residue may influence R-spine dynamics. We may thus suggest that T409 (in the β5-strand) might correspond to the gatekeeper residue in the predicted pUL97 kinase (Figure S9), which may explain why T409M mutation is the most frequently encountered mutation in the clinical setting [101].

H411L substitution confers a high level of resistance to MBV (69-fold increases in wild-type EC_50_), whereas H411N/Y induces moderate resistance to the drug (9- and 12-fold increases in wild-type EC_50_, respectively) [67]. The GCV susceptibility of recombinant viruses harboring these 3 amino acid substitutions was not affected compared to that of the wild type. The replicative capacity of the H411Y recombinant mutant virus was similar to that of the wild type [92]. As for T409M, the substitution of histidine (Figure 5C) with leucine (Figure 5D), asparagine (Figure 5E), or tyrosine (Figure 5F) may alter the shape of the cavity in the vicinity of K355 and may affect MBV docking.

L397R substitution induces a very high level of resistance to MBV (>200-fold increases in wild-type EC_50_), but not to GCV [91]. The replicative capacity of the recombinant L397R mutant virus was not affected compared to that of the wild type [91]. The substitution of leucine (Figure S10A) with bulky arginine (Figure S10B) at position 397 may alter the shape of the docking cavity of MBV in the vicinity of K355.

L337M substitution is located in the P-loop in the conserved structural sub-domain I. This amino acid substitution confers a low level of resistance to MBV (3.5-fold increases in wild-type EC_50_), but not to GCV [93]. The replicative capacity of the recombinant L337M mutant virus was not affected compared to that of the wild type [93]. The substitution of leucine displaying a bulky aliphatic side chain (Figure S10C) with methionine (Figure S10D) at position 337 may affect the environment of V345 and K355 residues, which may change the shape of the docking cavity of MBV in the vicinity of K355.

V353A substitution occurs in the ATP binding site (conserved structural sub-domain II) and confers moderate resistance to MBV (15-fold increases in wild-type EC_50_), but not to GCV [91]. This amino acid change does not affect the growth of the recombinant mutant virus compared to the wild type [91]. Although they are both aliphatic, the substitution of bulky valine (Figure S11A) with alanine (Figure S11B) at position 353 may disrupt the interaction with R413 and may affect the interaction with K355. Both changes may modify the shape of the docking cavity of MBV.

V356G substitution, which occurs in the ATP binding site, confers a very high level of resistance to MBV (108-fold increases in wild-type EC_50_) and cross-resistance to GCV (5.5-fold increases in wild-type EC_50_) [94]. The replicative capacity of the recombinant V356G mutant virus was decreased compared to that of the wild-type counterpart [94]. The substitution of bulky aliphatic valine (Figure S11C) with glutamine (Figure S11D) at position 356 may affect the adjacent K355 and the surrounding cavity. A change in the positioning of K355 may affect the interaction between the phosphate oxygen groups of ATP and the D481 residue, which might be involved in Mg^2+^ ions coordination. This may affect the activity of the enzyme, including the phosphorylation of GCV. The interaction with V354 may also alter the environment of V345 and the docking of MBV.

**Figure 6:**
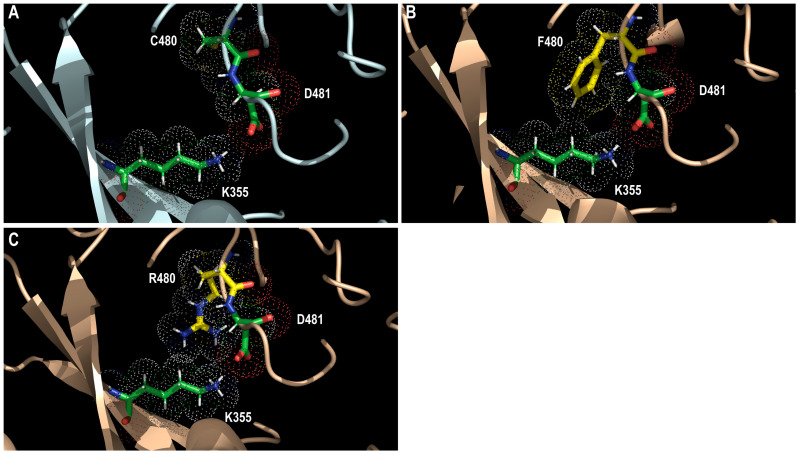
Structural representation of C480F and C480R amino acid substitutions. Wild-type (C480) predicted pUL97 structure (in pale cyan; (**A**)) and model of F480 (**B**) and R480 (**C**) mutants (wheat color). Wild-type C480 residue is shown using green sticks whereas mutated F480 and R480 residues are shown using yellow sticks. Residue K355 and D481 are also shown using green sticks. Dots show the atomic radius.

C480F/R substitutions were detected in clinical specimens collected from patients treated with MBV. These substitutions confer very high levels of resistance to MBV (224- and 243-fold increases in wild-type EC_50_, respectively) and GCV (2.3- and 9-fold increases in wild-type EC_50_, respectively) [95,96]. The viral growth of the recombinant C480F and C480R mutants was moderately and markedly reduced compared to that of the wild-type counterpart, respectively [95,96]. This cysteine, located in the putative catalytic site, is at the frontier between the two main clusters of mutations. The substitution of cysteine (Figure 6A) with bulky arginine (Figure 6B) or phenylalanine (Figure 6C) residue may alter the shape of the cavity in the vicinity of K355. Indeed, in contrast to C480, F480 and R480 may interact with the invariant K355, which may alter the docking cavity of MBV. C480 may establish hydrophobic contacts with MBV. In addition, the introduction of a bulkier residue may affect the positioning of the adjacent D481, with which MBV may interact by hydrogen bond. D481 might also be involved in the coordination of Mg^2+^ ions. The introduction of a positively charged arginine may have a greater effect on D481 than the phenylalanine residue. This may explain the very high level of resistance to MBV conferred by these substitutions. The enzyme activity may also be disturbed, including the phosphorylation of GCV.

The second cluster includes amino acid substitutions D456N, P521L, V466G, and F342S/Y. D456N substitution was selected in vitro under CPV and confers a very high level of resistance to MBV (278-fold increases in wild-type EC_50_) and to GCV (12-fold increases in wild-type EC_50_) [96]. A recombinant virus harboring this amino acid substitution showed a markedly reduced replicative capacity compared to the wild type [96]. The substitution of aspartic acid (Figure S12A) with asparagine (Figure S12B) at position 456 may alter the phosphoryl transfer reaction. According to docking results, D456 may be involved in hydrogen bonding with the ribose group of MBV and with the isopropylamino group of GCV. Changing the D456 residue may thus result in MBV and GCV resistance and alter the phosphorylating activity of the enzyme.

P521L substitution was found in clinical specimens obtained from patients treated with GCV. This amino acid change is located in the putative catalytic site and confers a very high level of resistance to MBV (428-fold increases in wild-type EC_50_) and GCV (17-fold increases in wild-type EC_50_) [94]. The recombinant P521L mutant exhibits a markedly altered viral growth compared to the wild type [94]. The substitution of proline (Figure S12C) by leucine (Figure S12D) may alter the interaction with Y519, which may affect the positioning of D456 and N461. Changes in the environment of the catalytic D456 residue and N461 (which might be involved in metal ion coordination) may result in MBV and GCV resistance, and may also alter the phosphorylating activity of the enzyme.

V466G substitution was detected in a clinical specimen from a patient treated with GCV [97]. V466G substitution confers a very high level of resistance to MBV (321-fold increases in wild-type EC_50_) and to GCV (11-fold increases in wild-type EC_50_) [94]. Recombinant virus with V466G substitution exhibits a significant growth defect that results in abnormal cytopathic effects similar to those observed with the pUL97 defective mutant [94,97]. The substitution of bulkier aliphatic valine (Figure S12E) with glutamine (Figure S12F) at position 466 may disrupt the interaction with H420, which may alter the positioning of F419, M460, N461, and D456. F419 may establish hydrophobic contact with MBV. The displacement of D456 may alter the binding of MBV and GCV. Changes in the environment of the catalytic D456 and N461 may affect the phosphorylating activity of the enzyme.

**Figure 7:**
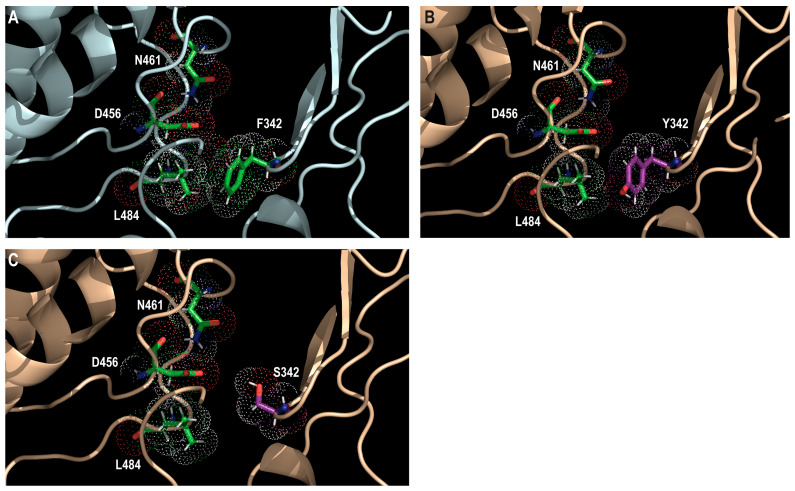
Structural representation of F342Y and F342S amino acid substitutions. Wild-type (F342) predicted pUL97 structure (in pale cyan; (**A**)) and models of Y342 (**B**) and S342 (**C**) mutants (wheat color). Wild-type F342 residue is shown using green sticks, whereas mutated Y342 and S342 residues are shown using magenta sticks. Residues L484, D456, and N461 are also shown using green sticks. Dots show atomic radius.

F342S/Y substitutions are located in the P-loop in the conserved structural sub-domain I. F342Y substitution confers a low level of resistance to MBV (4.7-fold increases in wild-type EC_50_) and GCV (6-fold increases in wild-type EC_50_) [68]. The less conservative F342S substitution confers a moderate level of resistance to MBV (18-fold increases in wild-type EC_50_) and GCV (7.8-fold increases in wild-type EC_50_) [94]. The substitution of phenylalanine (Figure 7A) by tyrosine (Figure 7B) or serine (Figure 7C) at position 342 may alter the positioning of L484, D456, and N461. Changes in the positioning of D456 may alter the binding of MBV and GCV. The replicative capacity of recombinant F342Y mutant was not affected compared to that of the wild-type virus [68], whereas that of F342S was moderately reduced [94]. The less conservative serine residue may further affect the environment of the catalytic D456 and N461, which are involved in the phosphorylating activity of the enzyme.

### 3.6. Amino Acid Substitutions Conferring Hyperscuceptibility to Maribavir

V345I, M460I, and M460V substitutions were detected in specimens of patients treated with GCV. V345I substitution (in the P-loop) does not affect susceptibility to GCV, whereas M460I and M460V substitutions (in the putative catalytic site) confer resistance to this drug (12.0- and 9.1-fold increases in GCV EC_50_, respectively) [99,100]. These three amino acid changes were also shown to induce hypersusceptibility to MBV (0.4-, 0.2- and 0.3-fold increases in wild-type EC_50_, respectively) [91,98,99]. The comparison of V345I mutant protein with the wild-type pUL97 kinase revealed that the substitution of the valine (Figure S13A) with an isoleucine (Figure S13B) at position 345 may decrease the distance between the mutated amino acid and MBV, which may affect the establishment of hydrophobic contacts. The substitution of methionine (Figure S13C) with isoleucine (Figure S13D) or valine (Figure S13E) at position 460 may increase the number of hydrogen bonds that may be established between both mutated residues and MBV. Thus, these data suggest that V345I, M460I, and M460V amino acid substitutions may modulate CMV susceptibility to MBV by increasing drug binding or by another mechanism.

This study has some limitations. We acknowledge that AI systems such as AlphaFold2 do not take into account the binding of ligands, co-factors, or other proteins, covalent modifications, post-translational modifications, conformational changes, or environmental factors that may affect the protein structure. We also acknowledge that metal ions and water molecules, which may influence ATP and drug binding, are lacking in our docking experiments. Nevertheless, in the absence of a crystal structure of pUL97 kinase, the predictive model based on AI, with an active site in a functional conformation where the phosphate groups of ATP are configured for catalysis and the substrate phsphorylation site is accessible, could help us to gain further insights into the mechanisms of antiviral drug resistance. This model could also be used to guide the design of novel antiviral drugs. Apart from pUL97 kinase, MBV can also induce compensatory mutations in pUL27, but these mutations are less clinically relevant [103].

## 4. Conclusions

To our knowledge, this is the first report describing the use of the AI system, AlphaFold2, to predict the structure of the viral pUL97 kinase to evaluate the mechanisms of maribavir-induced inhibition and drug resistance mutations. By using the predicted pUL97 protein structure, with an active site in a functional conformation, docking experiments predicted that MBV is a dual-site ATP binding and substrate phosphorylation inhibitor. Substitutions conferring resistance to MBV only may directly or indirectly alter the shape of the cavity in the vicinity of the invariant K355 in the putative ATP binding site. In particular, the T409M substitution might correspond to a mutation at a gatekeeper residue as described for ATP binding inhibitors of cellular protein kinases. These substitutions may affect MBV binding to pUL97 kinase, but do not alter the viral replicative capacity. In contrast, substitutions inducing cross-resistance to MBV and GCV may directly or indirectly affect the environment of D456 and N461 residues in the putative catalytic site and may be similar to mutations induced by substrate phosphorylation site inhibitors of cellular protein kinases. These substitutions affect the viral growth and may alter ATP binding and the phosphorylating activity of the enzyme. These results have important implications with respect to the clinical use of MBV, which is indicated for the treatment of CMV infections that do not respond to (val)ganciclovir, foscarnet, or cidofovir. These data also suggest that other viral targets should be used to develop new antiviral drugs (e.g., inhibitors of the viral terminase complex such as letermovir). Our predicted model may also be of interest for the design of novel pUL97 kinase inhibitors.

## Figures and Tables

**Table 1:** Characteristics of recombinant viruses, harboring amino acid substitutions that confer drug resistance, analyzed in this study.

Amino Acid Changes	Fold Change Maribavir	Fold Change Ganciclovir	Viral Growth	Clinical/Lab-derived	Structural Representation *	References
**First cluster of amino acid substitutions conferring drug resistance**						
T409M	81×	0.9×	No change	Clinical	Figure 5	[91]
H411L	69×	0.7×	ND	Clinical	Figure 5	[67]
H411N	9×	1.0×	ND	Clinical	Figure 5	[67]
H411Y	12×	0.5×	No change	Clinical	Figure 5	[67,92]
L397R	200×	1.6×	No change	Lab-derived	Figure S10	[91]
L337M	3.5×	1.0×	No change	Lab-derived	Figure S10	[93]
V353A	15×	1.0–1.5×	No change	Lab-derived	Figure S11	[91]
V356G	108×	5.5×	Moderately decreased	Lab-derived	Figure S11	[94]
C480F	224×	2.3×	Moderately decreased	Clinical	Figure 6	[95]
C480R	243×	9.0×	Markedly decreased	Clinical	Figure 6	[96]
**Second cluster of amino acid substitutions conferring drug resistance**						
D456N	278×	12×	Markedly decreased	Lab-derived	Figure S12	[96]
P521L	428×	17×	Markedly decreased	Clinical	Figure S12	[94]
V466G	321×	11×	Markedly decreased	Clinical	Figure S12	[94,97]
F342Y	4.5×	6×	No change	Clinical	Figure 7	[68]
F342S	18×	7.8×	Moderately decreased	Lab-derived	Figure 7	[94]
**Amino acid substitutions conferring hypersusceptibility to maribavir**						
V345I	0.4×	1.3×	ND	Clinical	Figure S13	[98]
M460I	0.2×	12×	ND	Clinical	Figure S13	[99,100]
M460V	0.3×	9.1×	No change	Clinical	Figure S13	[91,92,100]

EC_50_: the effective concentration of antiviral drugs that reduces the viral growth by 50%. Fold changes are calculated as the ratio of mutant EC_50_ to wild-type EC_50_ values. ND: not determined. * Structural representations of amino acid substitutions that were detected in clinical specimens of patients treated with maribavir are shown in the main manuscript, whereas those that were selected in vitro under maribavir or another drug (such as cyclopropavir or a carbocyclic analog of maribavir) or detected in samples of patients treated with ganciclovir are shown in Supplementary Figures.

## Data Availability

The original contributions presented in this study are included in the article/Supplementary Materials. Further inquiries can be directed to the corresponding authors.

## Supplementary Materials

The following supporting information can be downloaded at: https://www.mdpi.com/article/10.3390/v17070941/s1, Figure S1: Multiple sequence alignment per residue of pUL97 kinase sequence. Figure S2: Amino acids of the predicted pUL97 kinase that might be involved in the binding of phosphate moiety of ATP and in the coordination of the metal ions. Figure S3: Structural representation of adenosine triphosphate and antiviral drugs docked to the predicted pUL97 kinase. Figure S4: Interaction diagrams of ATP and antiviral drugs to the predicted pUL97 kinase. Figure S5: Structural representation of pUL97 kinase with ATP and two Mg^2+^ ions predicted with AlphaFold3. Figure S6: Most probable poses of maribavir and ganciclovir to predicted pUL97 kinase. Figure S7: Localization of residues 379–382 relative to ligand docking sites in the predicted pUL97 kinase. Figure S8: Localization of the LxCxE motif in the predicted pUL97 kinase and structural representation of C151G amino acid substitution. Figure S9: Structural representation of the suggested regulatory spine and gatekeeper residue in the predicted pUL97 kinase. Figure S10: Structural representation of L397R and L337M amino acid substitutions. Figure S11: Structural representation of V353A and V356G amino acid substitutions. Figure S12: Structural representation of D456N, P521L and V466G amino acid substitutions. Figure S13: Structural representation of V345I, M460I and M460V amino acid substitutions. Table S1: Quality assessment of the different mutant pUL97 protein models. Supplementary files: atomplddt score_pUL97_kinase.PDF; predicted_aligned_error_pUL97_kinase.JSON.

## Abbreviations

The following abbreviations are used in this manuscript:

a.a.	Amino acid
AI	Artificial intelligence
ATP	Adenosine triphosphate
cAPK	Cyclic AMP-dependent protein kinase
CDK	Cyclin-dependent kinase
CMV	Cytomegalovirus
CPV	Cyclopropavir
EC_50_	Effective concentration of antiviral that reduces the viral growth by 50%
GCN2	General control kinase 2
GCV	Ganciclovir
GLIDE	Grid-based ligand docking with energetics
K_i_	Inhibition constant
I-TASSER	Iterative threading assembly refinement
MBV	Maribavir
MSA	Multiple sequence alignment
NEC	Nuclear egress complex
NLS	Nuclear localization signal
PAE	Predicted aligned error
pLDDT	Predicted local distance difference test
rb	Retinoblastoma
RS	Regulatory spine
SH	Shell
SLiM	Short linear motif
SRPK1	Serine–arginine protein kinase 1
