# Supplementary material for: In Silico Analysis of Mechanisms of Maribavir-Induced Inhibition and Drug Resistance Mutations in pUL97 Kinase Structural Prediction with AlphaFold2
Source: Viruses. 2025 Jul 2;17(7):941. doi: 10.3390/v17070941 (PMC12301049; doi:10.3390/v17070941)
Supplement: Supplementary file 1 [file viruses-17-00941-s001.zip › viruses-3708093-supplementary.pdf]

Supplementary materials

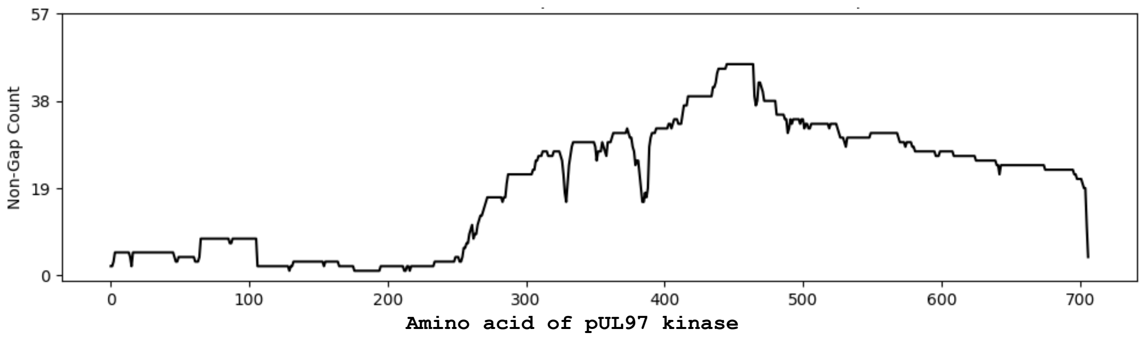

Figure S1. Multiple sequence alignment per residue of pUL97 kinase sequence. pUL97 kinase is composed of 707 amino acids. Prediction of protein structure was performed using AlphaFold2 [1,2].

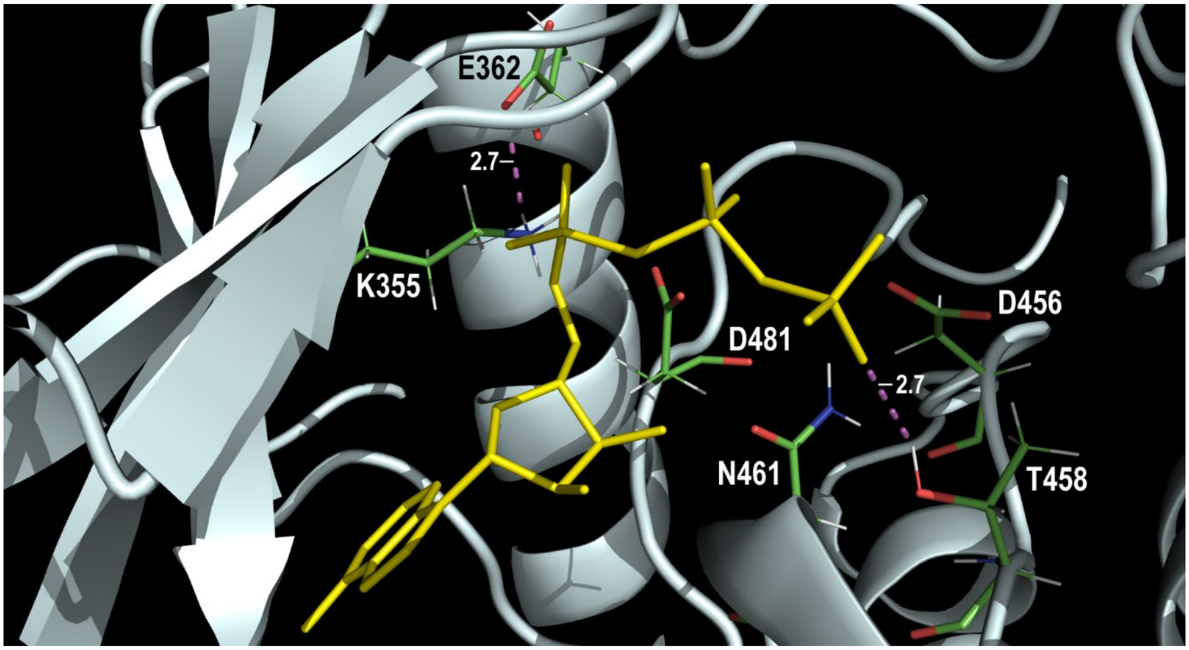

Figure S2. Amino acids of the predicted pUL97 kinase that might be involved in the binding of the phosphate moiety of ATP and in the coordination of the metal ions. pUL97 protein structure predicted using AlphaFold2 [1,2] is shown in pale cyan. The E362 residue in the αC-helix may interact with the invariant K355 residue. The K355 residue should be involved in the binding of α- and β-phosphate oxygen groups of ATP. The T458 residue may stabilize the γ-phosphate of ATP and the catalytic D456 is positioned for the phosphoryl transfer reaction. The D481 and N461 residues might be involved in the coordination of the Mg^{2+} ions. ATP molecule docked to pUL97 kinase using Maestro software version 14.3.129 (Schrödinger, LLC, New York, NY, USA) [3] is shown in yellow sticks. Protein structure with ATP bound were drawn using PyMOL molecular visualization software version 3.1.3 [4].

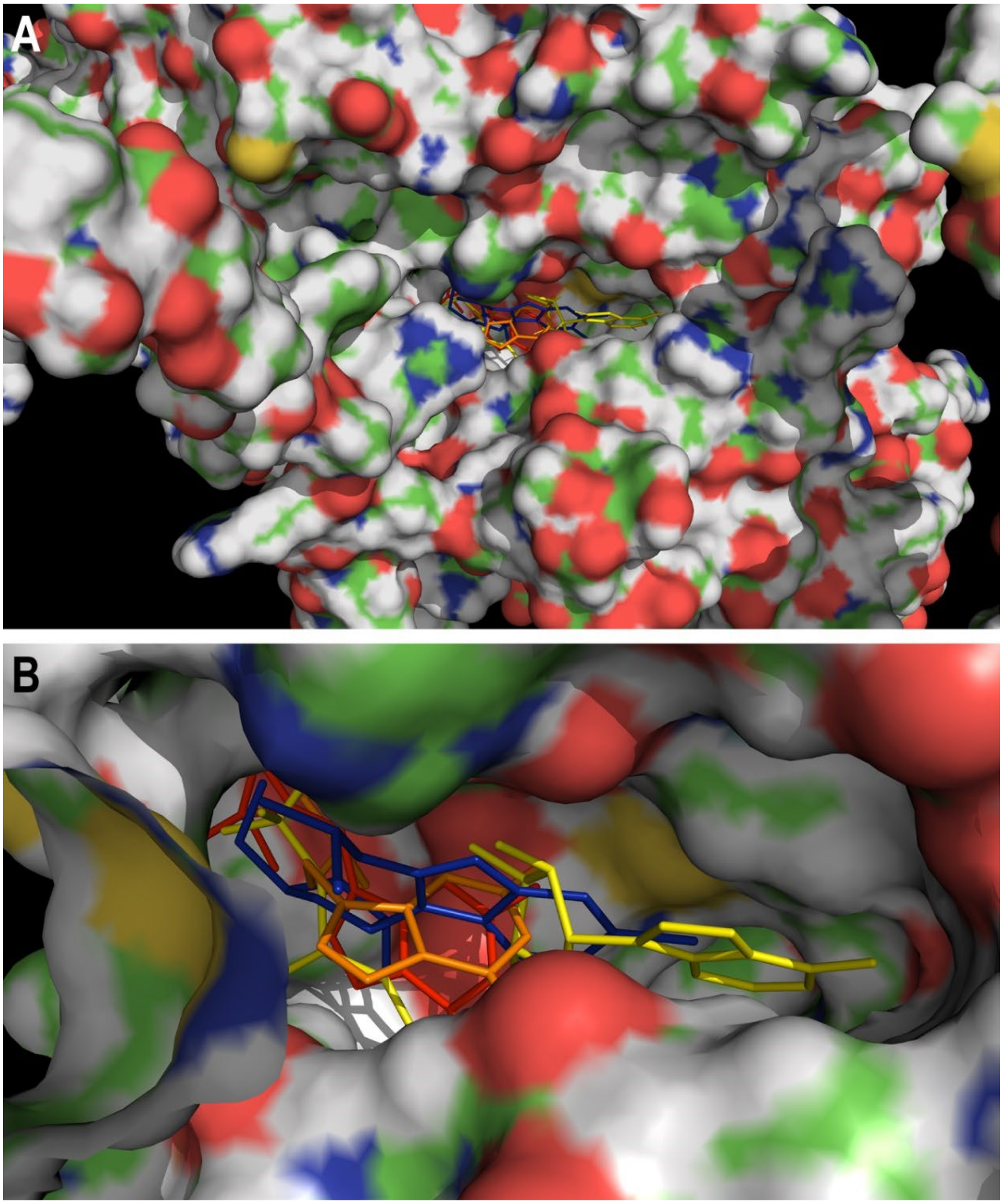

Figure S3. Structural representation of ATP and antiviral drugs docked to the predicted pUL97 kinase. A) ATP, maribavir, ganciclovir and cyclopropavir are located in the cleft between the amino-terminal lobe and the carboxy-terminal lobe of the predicted pUL97 kinase. ATP, maribavir, ganciclovir and cyclopropavir are shown using yellow, blue, orange and red sticks, respectively. B) Close up view of ATP and antiviral drugs docked to pUL97 kinase. Docking of ATP and antiviral drugs to predicted pUL97 protein structure was performed using Maestro software version 14.3.129 (Schrödinger, LLC, New York, NY, USA)[3]. Protein structure with the different ligands were drawn using PyMOL molecular visualization software version 3.1.3 [4].

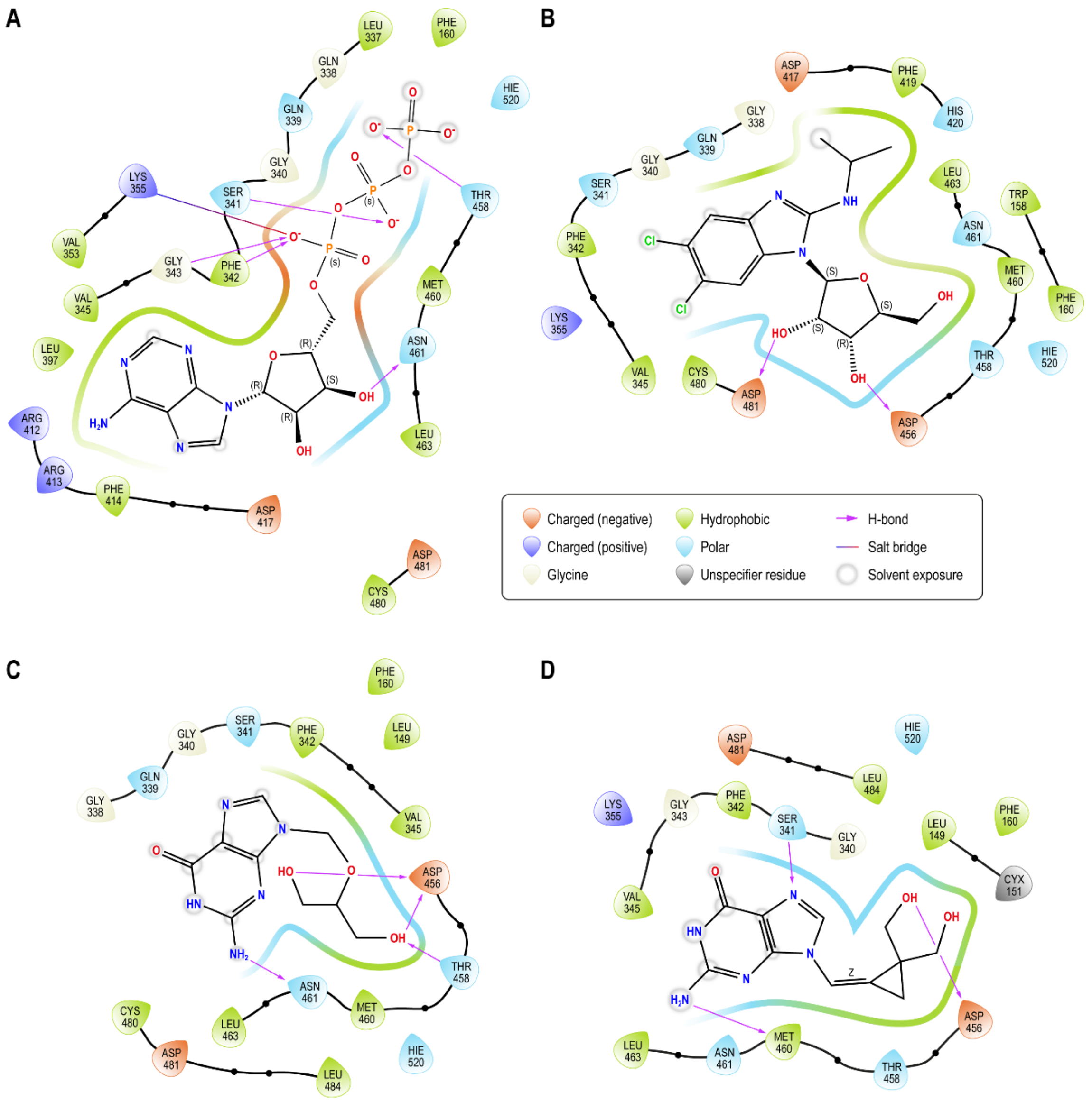

Figure S4. Interaction diagrams of ATP and antiviral drugs to the predicted pUL97 kinase. Residues of pUL97 kinase interacting with ATP (**A**), maribavir (**B**), ganciclovir (**C**) and cyclopropavir (**D**). Interactions diagrams were generated by Maestro software version 14.3.129 (Schrödinger, LLC, New York, NY, USA)[3].

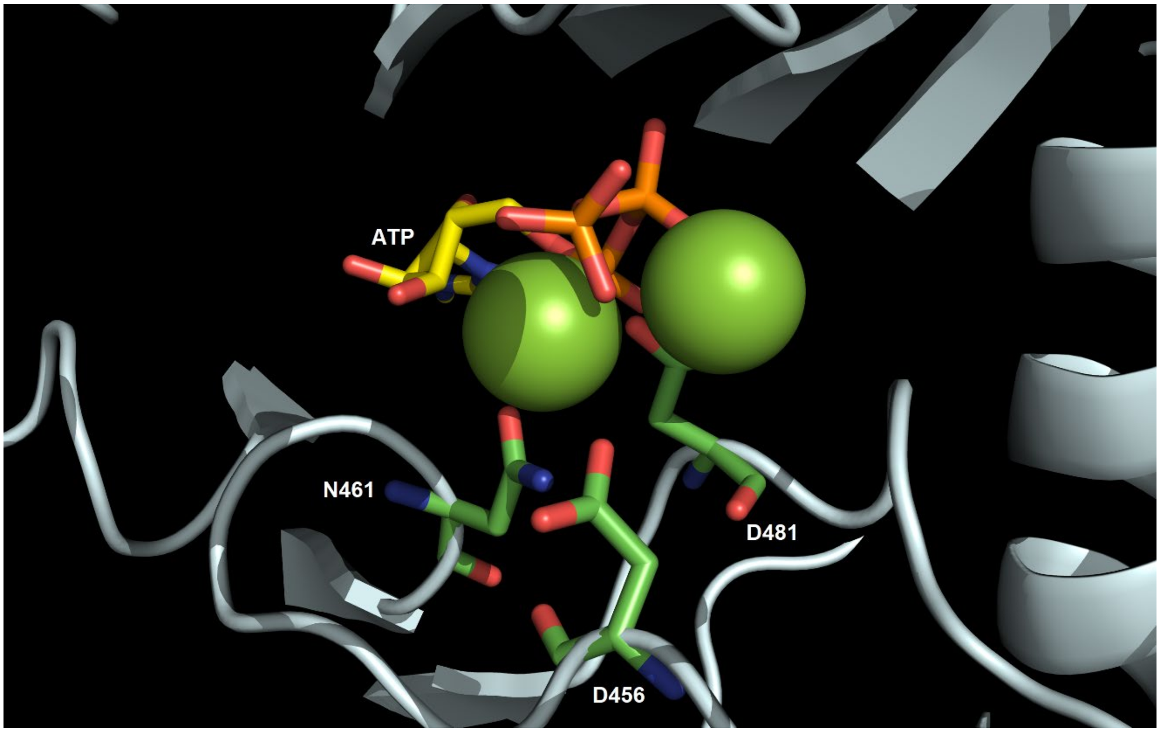

Figure S5. Structural representation of pUL97 kinase with ATP and two Mg^{2+} ions predicted with AlphaFold3. The left-handed Mg^{2+} ion may be coordinated by the α- and γ-phosphate oxygen groups of ATP and by the N461 and D481 residues (shown in green sticks). The right-handed Mg^{2+} ion may be coordinated by the β- and γ-phosphate oxygen groups of ATP and D481 residue. Mg^{2+} ions are represented using green spheres. The catalytic D456 residue is also shown using green sticks. The structure of pUL97 kinase with ATP and $Mg2^{+}$ ions was predicted using AlphaFold3 [5]. The predicted template modeling (pTM) score that measures the accuracy of the entire structure was estimated at 0.65 suggesting that the predicted fold might be similar to the true structure (pTM>0.5). The interface template modeling (ipTM) score that measures the accuracy of the predicted relative positions of the subunits within the complex was estimated to be 0.97 suggesting a confident high-quality prediction (ipTM>0.8). The root mean square deviation (RMSD) for the kinase domain (amino acids 281-707) of superimposed pUL97 structures predicted by AlphaFold2 and Alphafold3 was estimated to be 0.394 Å using PyMOL software Version 3.1.3 [4]. Divergences between the two predicted protein structures were observed in the disordered regions (amino acids 1-280). The protein structure with ATP and Mg^{2+} ions were drawn using PyMOL molecular visualization software version 3.1.3 [4].

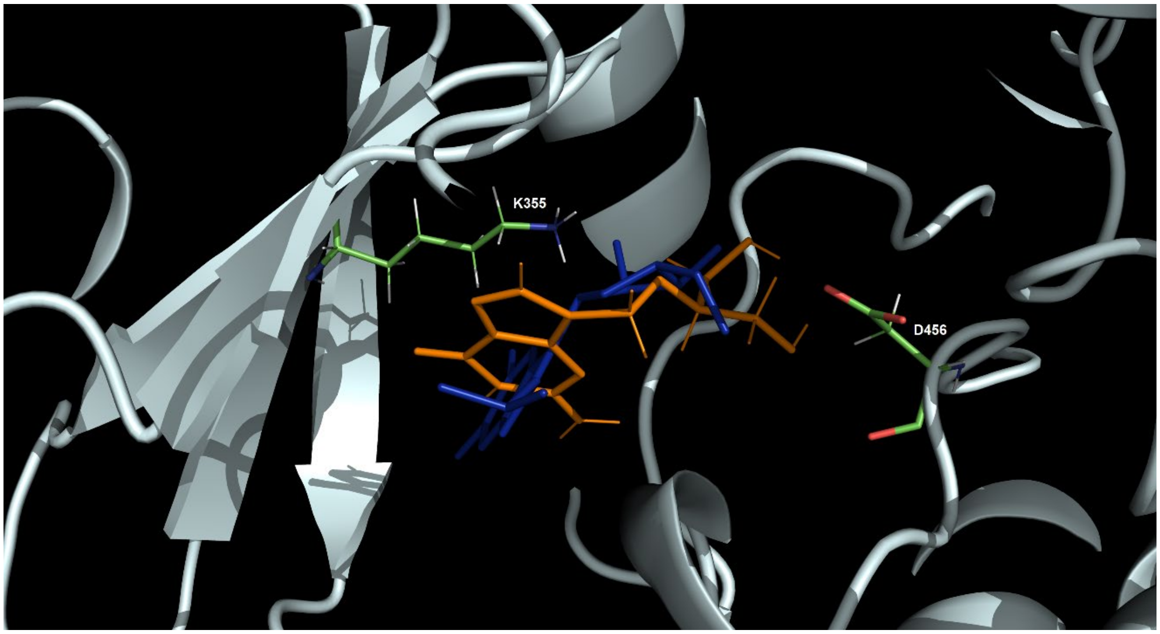

Figure S6. Most probable poses of maribavir with ganciclovir to predicted pUL97 kinase. Maribavir and ganciclovir were docked separately using Maestro software version 14.3.129 (Schrödinger, LLC, New York, NY, USA)[3] and were then superimposed to the predicted pUL97 protein structure (in pale cyan). The figure shows the clash between maribavir (shown using blue sticks) and ganciclovir (shown using orange sticks) molecules. The catalytic D456 and the invariant K355 residues are also shown using green sticks. The protein structure with maribavir and ganciclovir were drawn using PyMOL molecular visualization software version 3.1.3 [4].

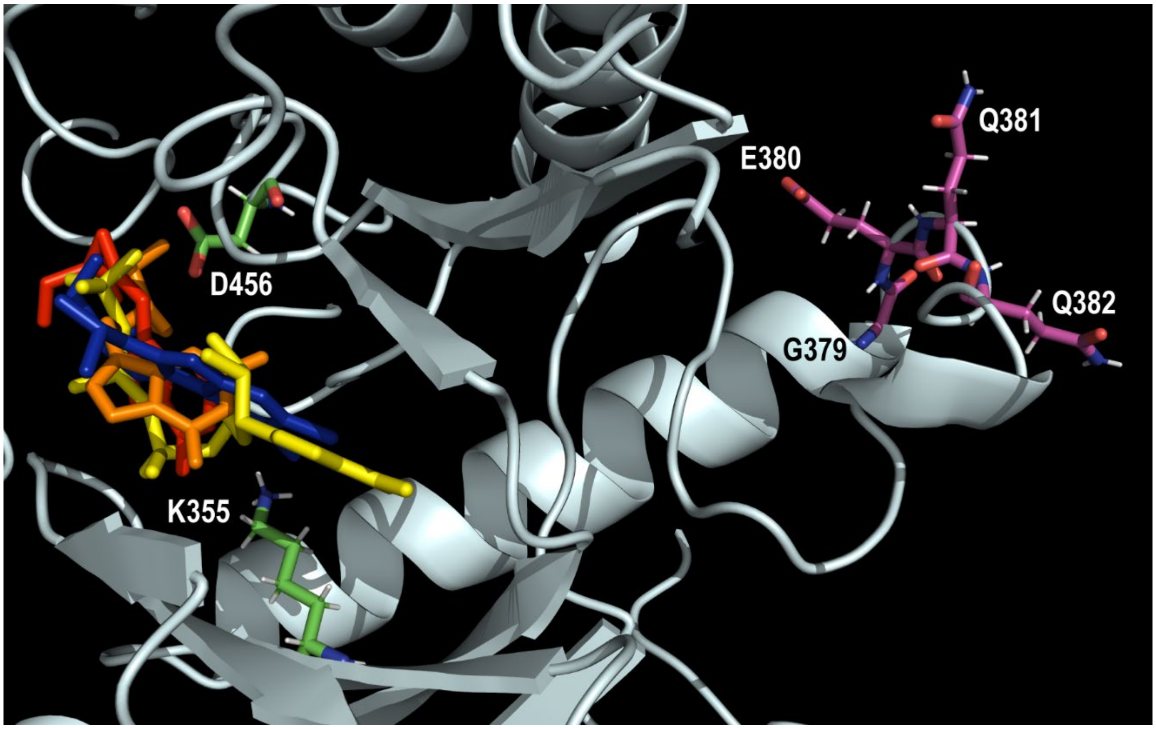

Figure S7. Localization of residues 379-382 relative to ligand docking sites in the predicted pUL97 kinase. The pUL97 protein structure is shown in pale cyan. Amino acids 379-382, which were predicted with a lower confidence level in the catalytic domain of pUL97 kinase are shown using magenta sticks. The most probable poses of ATP, maribavir, ganciclovir and cyclopropavir superimposed to the protein structure are shown using yellow, blue, orange and red sticks, respectively. The catalytic D456 and the invariant K355 residues are also shown using green sticks. Docking of ATP and antiviral drugs to predicted pUL97 protein structure was performed separately using Maestro software version 14.3.129 (Schrödinger, LLC, New York, NY, USA)[3]. Protein structures with the different ligands were drawn using PyMOL molecular visualization software version 3.1.3 [4].

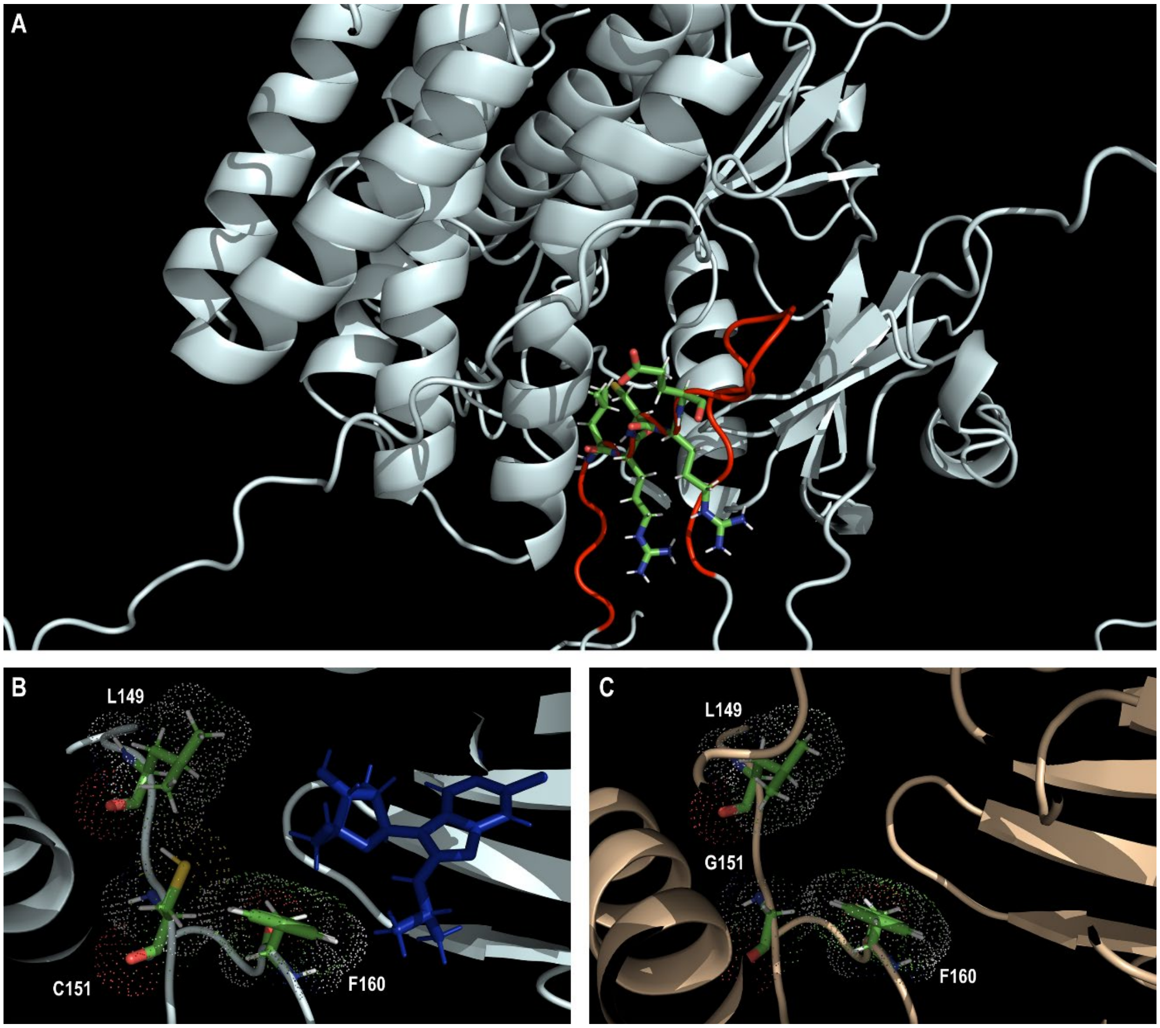

Figure S8. Localization of the LxCxE motif in the predicted pUL97 kinase and structural representation of C151G amino acid substitution. pUL97 protein structure (in pale cyan) with residues 145 to 165 (colored in red) and the LRCRE motif (shown using green sticks) (**A**). Wild type (C151) predicted protein structure (in pale cyan; **B**) and model G151 mutant (in wheat color; **C**). L149 and F160 residues are also shown using green sticks. Dots show the atomic radius. Maribavir is shown using blue sticks. Protein structure was drawn using PyMOL molecular visualization software version 3.1.3 [4].

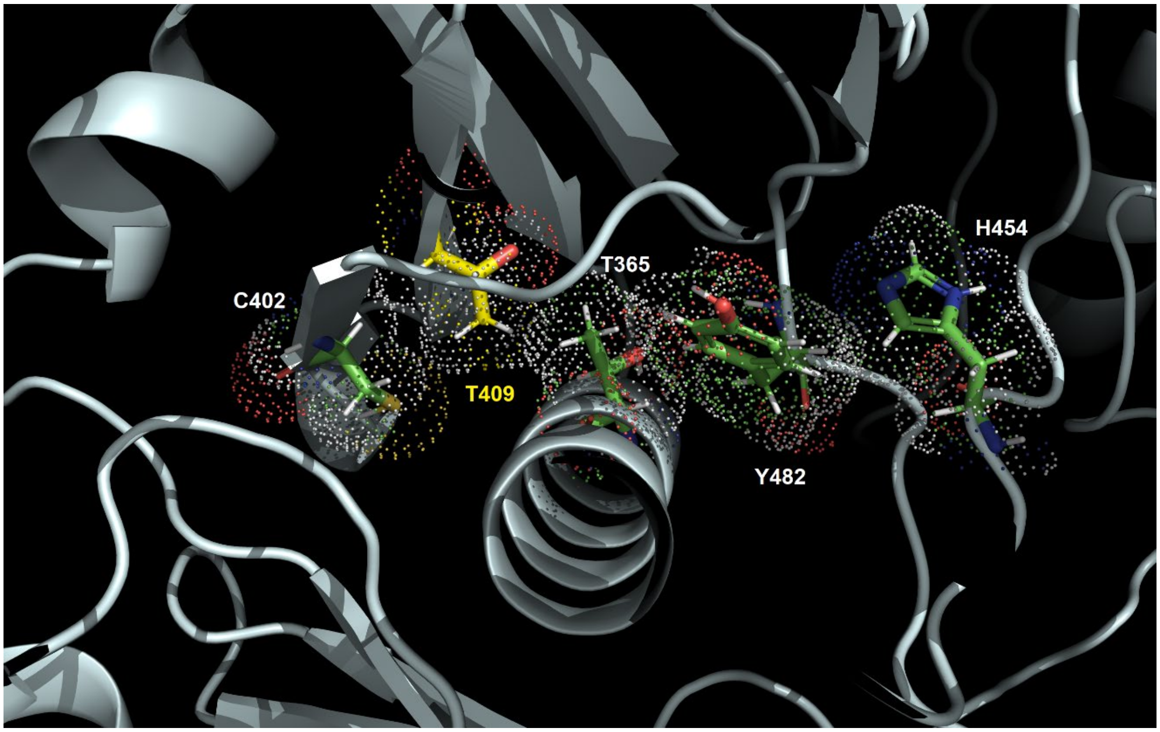

Figure S9. Structural representation of the suggested regulatory spine and gatekeeper residue in the predicted pUL97 kinase. pUL97 protein structure (in pale cyan) showing the 4 residues of the regulatory spine (SR1, H454 of the 454-HFD-456 motif; SR2, Y482 of the 481-DYS-483 motif; SR3, T365 in the αC-helix; SR4, possibly C402 in the β4-strand) identified by sequence homology with other protein kinases as described in [6] are shown using green sticks. The suggested gatekeeper residue T409 (in the β5-strand) is shown using yellow sticks. Dots show the atomic radius. Protein structure was drawn using PyMOL molecular visualization software version 3.1.3 [4].

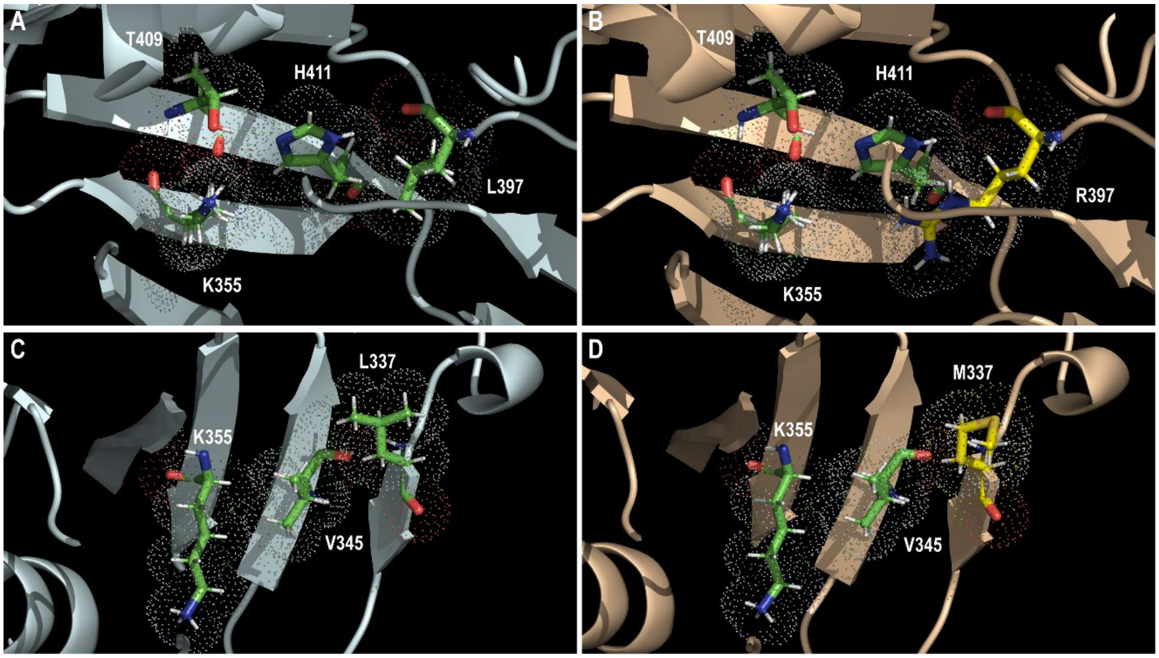

Figure S10. Structural representation of L397R and L337M amino acid substitutions. Wild type (L397) predicted pUL97 structure (in pale cyan; **A**) and model of R397 mutant (in wheat color; **B**). Wild type L397 residue is shown using green sticks whereas mutated M337 residue is shown using yellow sticks. Residues T409, H411 and K355 are also shown using green sticks. Wild type (L337) predicted pUL97 structure (in pale cyan; **C**) and model of M337 (in wheat color; **D**). Wild type L337 residue is shown using green sticks whereas mutated M337 residue is shown using yellow sticks. Residues V345 and K355 are also shown using green sticks. Dots show the atomic radius. Protein structures were drawn with PyMOL molecular visualization software version 3.1.3 [4].

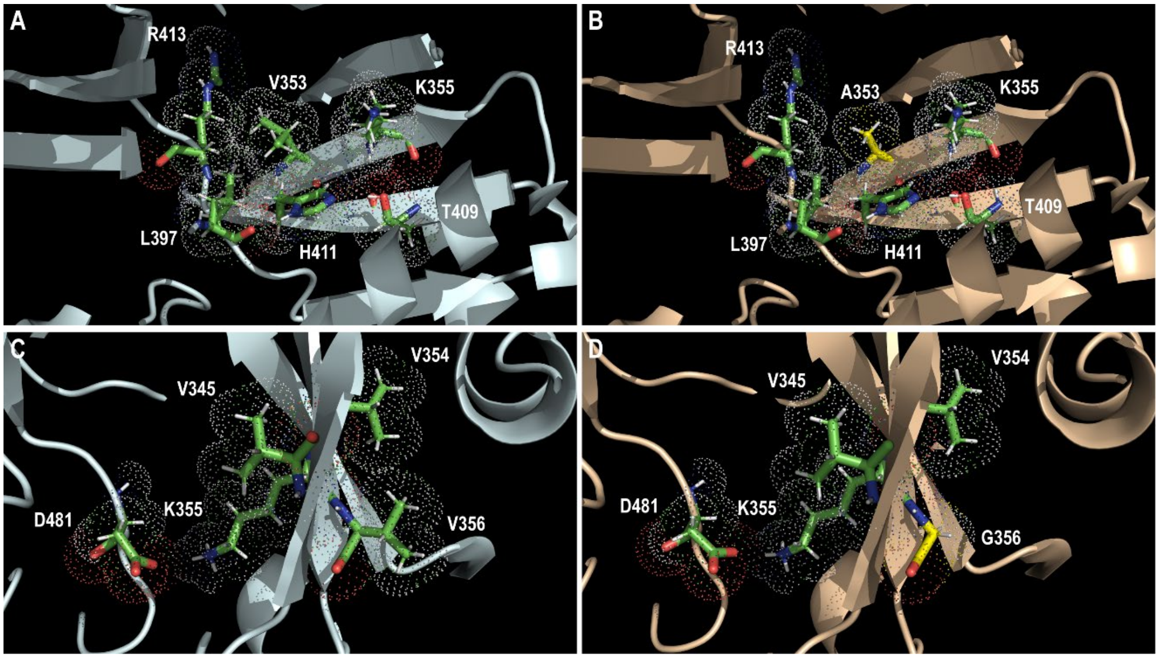

Figure S11. Structural representation of V353A and V356G amino acid substitutions. Wild type (V353) predicted pUL97 structure (in pale cyan; **A**) and model of A353 mutant (in wheat color; **B**). Wild type V353 residue is shown using green sticks whereas mutated A353 residue is shown using yellow sticks. Residues R413, L397, H411, T409 and K355 are also shown using green sticks. Wild type (V356) predicted pUL97 structure (in pale cyan; **C**) and model of G356 mutant (in wheat color; **D**). Wild type V356 residue is shown using green sticks whereas mutated G356 residue is shown using yellow sticks. Residues V354, V345, K355 and D481 are also shown using green sticks. Dots show the atomic radius. Protein structures were drawn with PyMOL molecular visualization software version 3.1.3 [4].

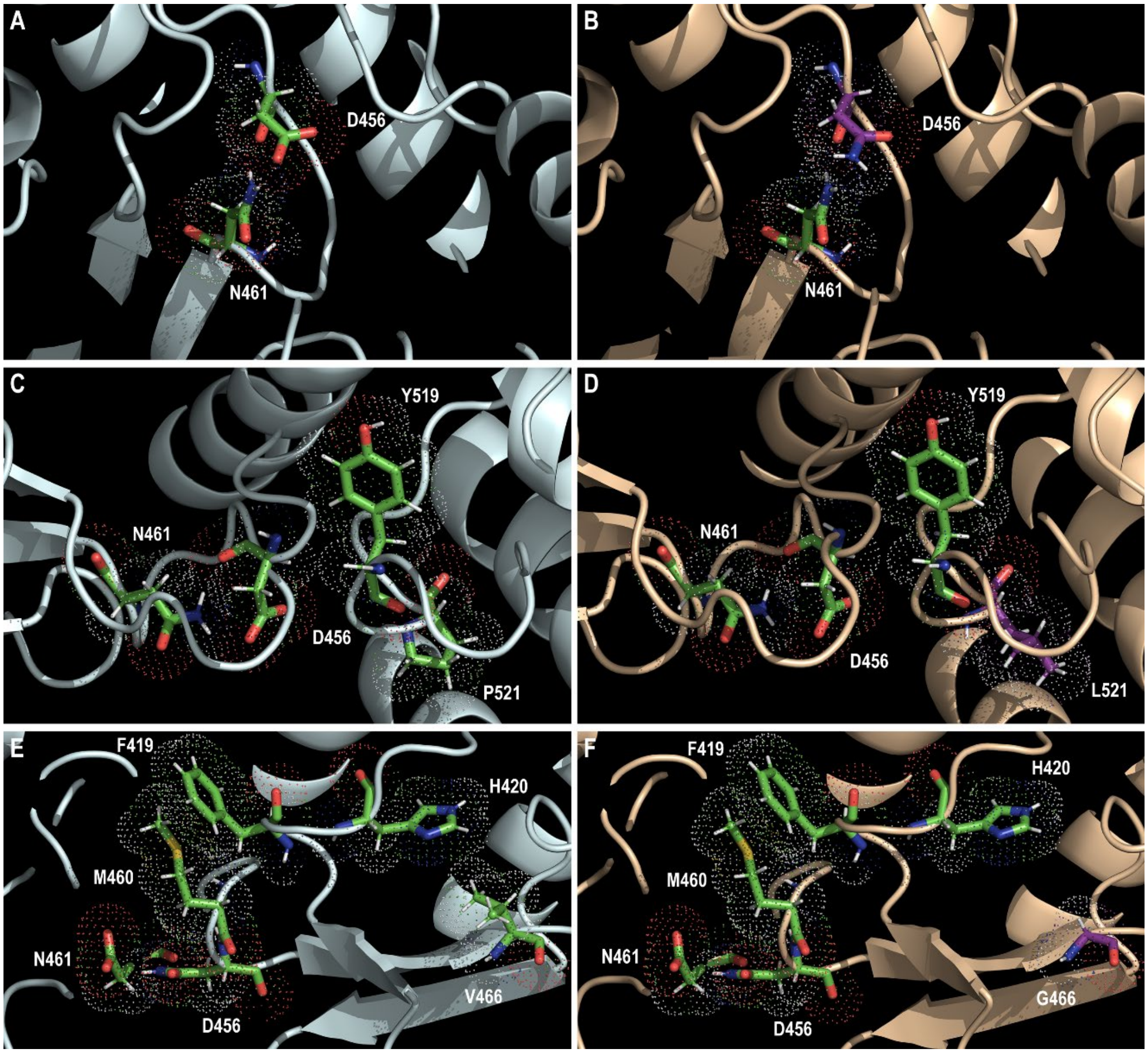

Figure S12. Structural representation of D456N, P521L and V466G amino acid substitutions. Wild type (D456) predicted pUL97 structure (in pale cyan; **A**) and model of N456 mutant (in wheat color; **B**). Wild type D456 residue is shown using green sticks whereas mutated N456 residue is shown using magenta sticks. Residue N461 is also shown using green sticks. Wild type (P521) (in pale cyan; **C**) and model of L521 mutant (in wheat color; **D**). Wild type P521 residue is shown using green sticks whereas L521 residue is shown using magenta sticks. Residues Y519, D456 and N461 are also shown using green sticks. Wild type (V466) predicted pUL97 structure (in pale cyan; **E**) and model of G466 mutant (in wheat color; **F**). Wild type V466 residue is shown using green sticks whereas mutated G466 residue is shown using magenta sticks. Residues H420, F419, M460, N461 and D456 are also shown using green sticks. Dots show the atomic radius. Protein structures were drawn with PyMOL molecular visualization software version 3.1.3 [4].

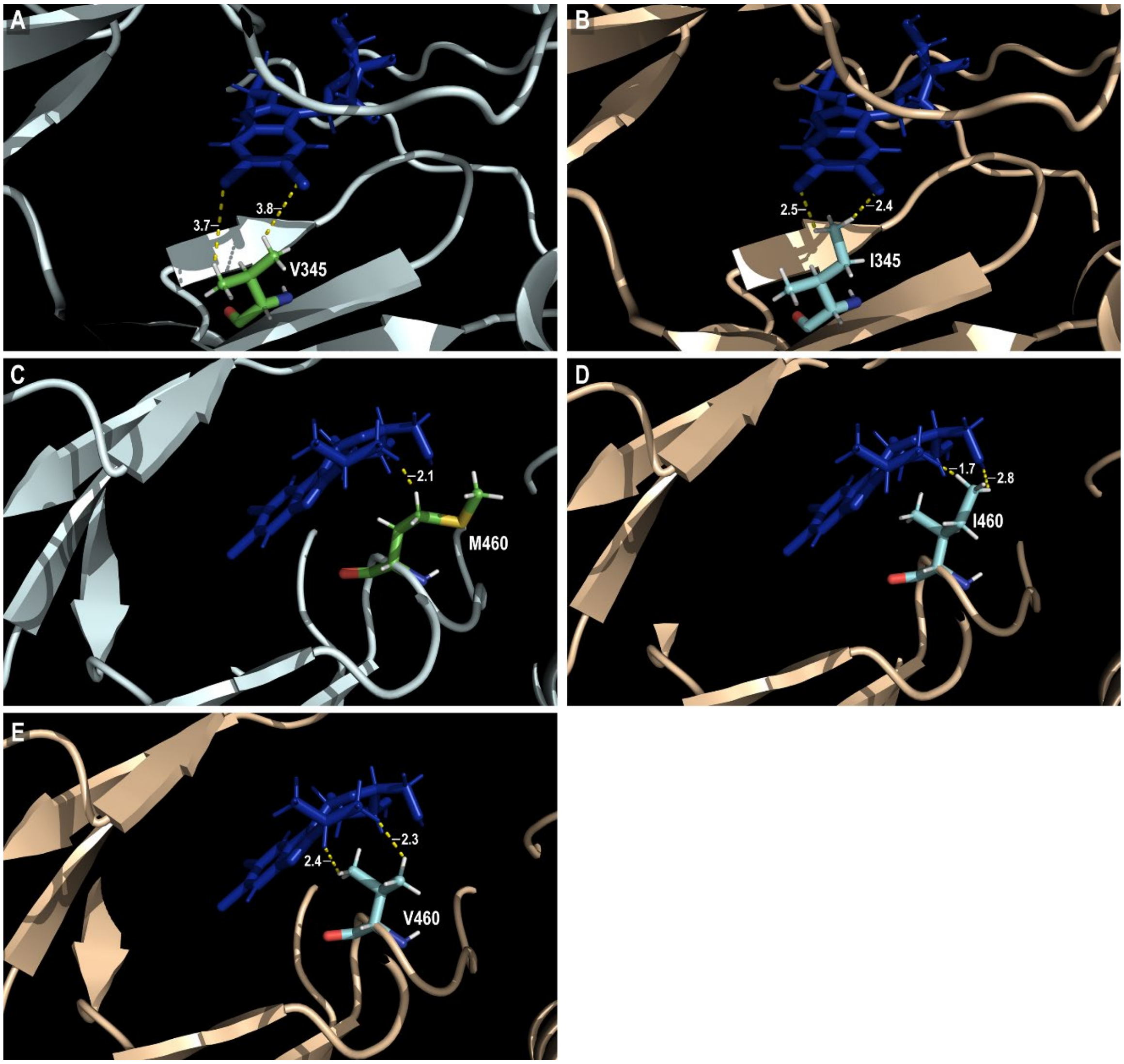

Figure S13. Structural representation of V345I, M460I and M460V amino acid substitutions. Wild type (V345) predicted pUL97 structure (in pale cyan; **A**) and model of I345 (in wheat color; **B**). Wild type V345 residue is shown using green sticks whereas mutated I345 residue is shown using light blue sticks. Wild type (M460) predicted pUL97 structure (in pale cyan) and models (in wheat color) of I460 (**D**) and V460 (**E**) mutants. Wild type M460 residue is shown using green sticks whereas mutated I460 and V460 residues are shown using light blue sticks. Dots represent the atomic radius. The distance between amino acid atoms that could affect the binding to maribavir (represented using dark blue sticks) are also shown. Protein structures were drawn with PyMOL molecular visualization software version 3.1.3 [4].

Table S1: Quality assessment of the different mutant pUL97 protein models.

Residue	Wild type pUL97	Mutant pUL97	RMSD (in Å)*	MolProbity score	Clash score
151	C	G	0.123	1.61	0.55
337	L	M	0.065	1.78	0.83
342	F	S	0.064	1.73	0.65
342	F	Y	0.064	1.78	0.83
345	V	I	0.064	1.78	0.83
353	V	A	0.064	1.78	0.83
356	V	G	0.064	1.78	0.83
397	L	R	0.065	1.78	0.83
409	T	M	0.065	1.78	0.83
411	H	L	0.064	1.78	0.83
411	H	N	0.064	1.78	0.83
411	H	Y	0.122	1.64	0.65
456	D	N	0.064	1.78	0.83
460	M	I	0.064	1.78	0.83
460	M	V	0.064	1.78	0.83
466	V	G	0.064	1.78	0.83
480	C	F	0.065	1.80	0.92
480	C	R	0.065	1.80	0.92
521	P	L	0.062	1.85	1.20

*, Root mean square deviation (RMSD) in Cα coordinates for the 707 residues of pUL97 kinase were determined using PyMOL molecular visualization software version 3.1.3 [4]. RMSD for wild type protein generated with Swiss Model [7] superimposed to wild type pUL97 protein predicted with AlphaFold2 is 0.064 Å. MolProbity and clash scores were determined using the MolProbity software version 4.5.2 [8,9].

References

1. Jumper, J.; Evans, R.; Pritzel, A.; Green, T.; Figurnov, M.; Ronneberger, O.; Tunyasuvunakool, K.; Bates, R.; Zidek, A.; Potapenko, A.; et al. Highly accurate protein structure prediction with AlphaFold. *Nature* **2021**, *596*, 583-589, doi:10.1038/s41586-021-03819-2.
2. Evans, R.; O'Neill, M.; Pritzel, A.; Antropova, N.; Senior, A.; Green, T.; Žídek, A.; Bates, R.; Blackwell, S.; Yim, J.; et al. Protein complex prediction with AlphaFold-Multimer. *bioRxiv* **2021**, 2021.2010.2004.463034, doi:10.1101/2021.10.04.463034.
3. Friesner, R.A.; Banks, J.L.; Murphy, R.B.; Halgren, T.A.; Klicic, J.J.; Mainz, D.T.; Repasky, M.P.; Knoll, E.H.; Shelley, M.; Perry, J.K.; et al. Glide: a new approach for rapid, accurate docking and scoring. 1. Method and assessment of docking accuracy. *Journal of medicinal chemistry* **2004**, *47*, 1739-1749, doi:10.1021/jm0306430.
4. Schrodinger, K.K. The PyMOL Molecular Graphics System, version 1.3rl. ; Schrödinger LLC: **2010**.
5. Abramson, J.; Adler, J.; Dunger, J.; Evans, R.; Green, T.; Pritzel, A.; Ronneberger, O.; Willmore, L.; Ballard, A.J.; Bambrick, J.; et al. Accurate structure prediction of biomolecular interactions with AlphaFold 3. *Nature* **2024**, *630*, 493-500, doi:10.1038/s41586-024-07487-w.
6. Shevchenko, E.; Pantsar, T. Regulatory spine RS3 residue of protein kinases: a lipophilic bystander or a decisive element in the small-molecule kinase inhibitor binding? *Biochem Soc Trans* **2022**, *50*, 633-648, doi:10.1042/BST20210837.
7. Waterhouse, A.; Bertoni, M.; Bienert, S.; Studer, G.; Tauriello, G.; Gumienny, R.; Heer, F.T.; de Beer, T.A.P.; Rempfer, C.; Bordoli, L.; et al. SWISS-MODEL: homology modelling of protein structures and complexes. *Nucleic acids research* **2018**, *46*, W296-W303, doi:10.1093/nar/gky427.
8. Williams, C.J.; Headd, J.J.; Moriarty, N.W.; Prisant, M.G.; Videau, L.L.; Deis, L.N.; Verma, V.; Keedy, D.A.; Hintze, B.J.; Chen, V.B.; et al. MolProbity: More and better reference data for improved all-atom structure validation. *Protein Sci* **2018**, *27*, 293-315, doi:10.1002/pro.3330.
9. Lovell, S.C.; Davis, I.W.; Arendall, W.B., 3rd; de Bakker, P.I.; Word, J.M.; Prisant, M.G.; Richardson, J.S.; Richardson, D.C. Structure validation by Calpha geometry: phi,psi and Cbeta deviation. *Proteins* **2003**, *50*, 437-450, doi:10.1002/prot.10286.
